# La_2–*x*_Sr_*x*_Ce_2–*y*_Ni_*y*_O_7_ Catalysts with Interstitial Nickel for Enhanced Dry Reforming of Methane

**DOI:** 10.1021/acsami.5c11404

**Published:** 2025-10-30

**Authors:** Aathira Bhaskaran, Naga Pranava Sree Kothoori, Pralok K. Samanta, Stéphane Loridant, Patrick Da Costa, Satyapaul A. Singh, Sounak Roy

**Affiliations:** † Department of Chemistry, 209298 Birla Institute of Technology and Science (BITS) Pilani, Hyderabad Campus, Hyderabad 500078, India; ‡ Department of Chemistry, School of Science, 641915 Gandhi Institute of Technology and Management (GITAM), Hyderabad 502329, India; § Université Claude Bernard Lyon 1, CNRS, IRCELYON, UMR 5256, Villeurbanne F-69100, France; ∥ Institut Jean le Rond d′Alembert, 517735 Sorbonne Université, CNRS UMR 7190, 2 Place de la Gare de ceinture, Saint Cyr l′Ecole 78210, France; ⊥ Department of Chemical Engineering, 209298 Birla Institute of Technology and Science (BITS) Pilani, Hyderabad Campus, Hyderabad 500078, India; # Materials Centre for Sustainable Energy and Environment (McSEE), Birla Institute of Technology and Science (BITS) Pilani, Hyderabad Campus, Hyderabad 500078, India

**Keywords:** CO_2_ utilization, methane, dry reforming, defective fluorite, interstitial nickel

## Abstract

This study explores the catalytic performance of solution combustion-synthesized doped defective fluorite catalysts, La_2–_ *_x_* Sr*_x_* Ce_2–_ *_y_* Ni*_y_* O_7_, for the dry reforming of methane. A comprehensive structural analysis, supported by theoretical calculations, revealed that the adopted synthetic methodology enabled Ni doping beyond a critical concentration, leading to its occupation of the interstitial lattice sites. The optimally doped Ni-containing defective fluorite oxide La_1.9_Sr_0.1_Ce_1.7_Ni_0.3_O_7_ exhibited superior catalytic activity with more than 70% conversion of CO_2_ and CH_4_ with an H_2_/CO ratio of 0.7 for a 50-h reaction at 700 °C. The prolonged reforming reaction also resulted in minimal coke deposition (11 μg_c_ g_cat_^–1^ h^–1^), primarily due to the oxidative dissociation pathway of methane, as revealed through mechanistic analysis. Detailed surface studies highlighted the crucial role of metal–support interactions, wherein facile electron transfer from Ni to Ce during the reaction contributed significantly to the enhanced catalytic performance. Thus, this study establishes a strategic framework for designing and developing defect-engineered oxide catalysts, paving the way for advanced materials in dry methane reforming.

## Graphical abstract

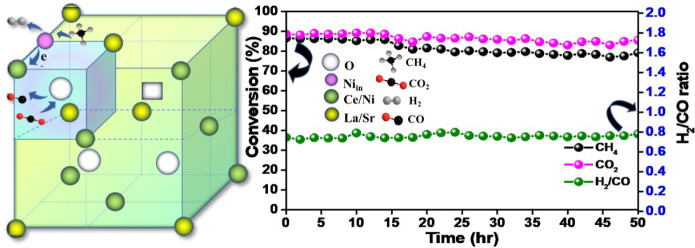

## 1 Introduction

Methane, a major component of natural gas emitted from various natural and anthropogenic sources, is one of the most abundant and cost-effective C_1_ feedstocks. However, its utilization poses a challenge due to the remote locations of methane reservoirs relative to industrial facilities, making transportation economically unfeasible or even impossible. Consequently, substantial amounts of gas are currently reinjected, flared, or vented in refineries, chemical plants, and oil wells. This practice contributes significantly to pollution by releasing both CO_2_ and unburned CH_4_ greenhouse gases into the atmosphere. An efficient, economical, and environmentally clean approach to methane utilization is its conversion into more valuable chemicals through synthesis gas (syngas, a mixture of H_2_ and CO) via the dry reforming of methane (DRM).^1–6^ The syngas produced is a suitable feedstock for long-chain hydrocarbon synthesis through the Fischer–Tropsch processes.^7^

The specific reaction involved in DRM is as follows:

1 $${\mathrm{CH}}_{4}+{\mathrm{CO}}_{2}\to 2H_{2}+2\mathrm{CO}(\Delta H_{298K}^{{}^{\circ}}=+247{\mathrm{kJ mol}}^{-1})$$

However, the inert symmetrical tetrahedral structure of CH_4_ makes it rather difficult for the dissociation of the first C–H bond, which is the most important step for the activation of methane.^8^ Also, owing to the highly endergonic nature of CO_2_ dissociation, a consequence of its high stability, its activation is extremely challenging. Furthermore, along with the dry reforming reaction, side reactions such as the reverse water–gas shift (RWGS) reaction (CO_2_ + H_2_ → CO + H_2_O), methane decomposition (CH_4_ ⇋ C_ad_ + 2H_2_), and the Boudouard reaction (2CO ⇌ C_ad_ + CO_2_) occur simultaneously, collectively reducing the H_2_/CO ratio and lowering DRM efficiency by promoting coke formation over the catalyst.

Therefore, a catalyst with adequate activity, selectivity, and stability is essential for DRM. An optimal DRM catalyst must possess heightened CH_4_ and CO_2_ activity, discerning H_2_ and CO selectivity in the right proportion, and exceptional stability against coke formation and metal sintering. While numerous combinations of metal active sites, supports, and promoters for DRM have been reported, achieving the synergistic effects of all these components within a single, integrated solid solution catalyst offers a novel and potentially more efficient approach to DRM.^9,10^ However, the high temperature in DRM sinters the supported dispersed active metal nanoparticles in conventional heterogeneous catalysts. It is necessary to develop stable materials that are catalytically active above 600 °C and resistant to carbon formation. To address the stability problem, coke resistance, as well as easy activation of both CH_4_ and CO_2_, different metal oxides with stable structures, such as perovskite, double perovskite, spinel, pyrochlore, etc., are presently attracting attention primarily due to their oxygen vacancy and redox couple availability as described as follows:

2 $$M_{x}O_{y}+{\delta \mathrm{CH}}_{4}\to M_{x}O_{y-\delta }+\delta \mathrm{CO}+{2\delta H}_{2}$$

3 $$M_{x}O_{y-\delta }+{\delta \mathrm{CO}}_{2}\to M_{x}O_{y}+\delta \mathrm{CO}$$

4 $${\delta \mathrm{CH}}_{4}+{\delta \mathrm{CO}}_{2}\to 2\delta \mathrm{CO}+{2\delta H}_{2}$$

Pyrochlores with A_2_B_2_O_7_ general formula are one such category of oxides that are not only resistant to sintering at high temperatures but also have improved oxygen vacancy and good oxygen mobility.^11–14^ A_2_B_2_O_7_ typically falls under the pyrochlore, defective fluorite, or perovskite category depending upon the Shannon radius ratio of the A and B cations, with the larger A-site cation typically 8-fold coordinated and the smaller B-site cation 6-fold coordinated, i.e., phase structure can be configured as defective fluorite if the *R*_A_/*R*_B_ ratio is <1.46, pyrochlore in the case of 1.46 < *R*_A_/*R*_B_ < 1.78 and if *R*_A_/*R*_B_ > 1.78 then it is regarded as perovskite.^15^ The defect fluorite structure can be expressed as A_2_B_2_O_6_O to distinguish the two different anion sites with O occupying the 48*f* Wyckoff sites at (*x*, 1/8, 1/8) (in space group *Fd*3̅*m*) and O occupying the 8*b* sites at (3/8, 3/8, 3/8). The two cations occupy special positions with the larger A-type cation at 16*d* (1/2, 1/2, 1/2) and the smaller B cation at 16*c* (0, 0, 0). Hence, the structure is described by the cubic lattice parameter and the positional parameter *x* for O. In this description, the 8*a* site at (1/8, 1/8, 1/8) is unoccupied, whereas in the (A/B)­O2–_*x*_ anion-deficient fluorite structure (space group *Fm*3̅*m*), the two cations are disordered at the 4*a* site (0, 0, 0), and the anions occupy the 8*c* site at (1/4, 1/4, 1/4).^16^ Noble metal-doped pyrochlores are a popular choice for the reaction because of their high activity and low coke formation.^17^ In one of the early studies, Ashcroft et al. concluded with the help of in situ energy-dispersive X-ray diffraction studies that between Eu_2_Ir_2_O_7_ and Eu_2_Ru_2_O_7_ pyrochlores, the iridates reduce directly to metallic iridium during DRM, while the ruthenates reduce to ruthenium via a stable, oxygen-deficient intermediate.^18^ Noble metals, such as Pt and Ru doped at B sites of La_2_Zr_2_O_7_ exhibited different mechanisms during DRM. The Pt-doped La_2_Zr_2_O_7_ apparently activated CH_4_ via formyl or formate group formation, whereas Ru in the B site activated CH_4_ via a direct decomposition route. For both pyrochlore catalysts, the mobile lattice oxygen was found to be reactive toward the carbon formed during DRM.^19,20^ However, despite their catalytic proficiency in DRM, noble metal catalysts are deemed impractical due to their elevated cost and restricted availability. Consequently, substantial research endeavors have been directed toward transition metal-based pyrochlore catalysts, which have demonstrated high efficacy in DRM while remaining cost-effective and abundantly accessible.^21^ The ionic doping of transition metal Ni in the B sites of A_2_B_2_O_6_O has shown promising DRM activity owing to the redox active Ni and oxygen mobility.^22^ La_2_Zr_2_O_7_ was synthesized with substitution on the B site with 1 and 6% Ni. Though the Ni-doped materials showed better DRM activity than unsubstituted pyrochlore, as the reaction proceeded, the Ni from the 6% Ni-doped La_2_Zr_2_O_7_ came out of the lattice as NiO and La_2_NiZrO_6_.^23^ Ramon et al. recently developed a mixed metal oxide system of La_2_Ce_2_O_7_ and LaNiO_3_.^12^ The metal oxides were reduced to obtain an *in situ* phase of La_2_Ni_2_O_5_ as the active site for the reaction. Due to intrinsic oxygen mobility, vacancy, and basicity, the catalyst showed high activity. The Pechini and modified hydrothermal methods of synthesis had the drawback of longer synthesis durations, resulting in high energy consumption.

In this work, a series of ternary defective fluorite La_2–*x*_Sr_*x*_Ce_2–*y*_Ni_*y*_O_7−δ_ (*x* = 0, 0.1; *y* = 0, 0.1, 0.3, 0.5) catalysts, synthesized via a one-step solution combustion method, were investigated for DRM with a detailed mechanistic analysis. Structural studies revealed that the typical synthesis procedure directed doped Ni to occupy interstitial positions within the lattice after a certain concentration. DRM activity tests demonstrated that the optimally doped compositions, La_1.9_Sr_0.1_Ce_1.7_Ni_0.3_O_7_ and La_1.9_Sr_0.1_Ce_1.5_Ni_0.5_O_7_, exhibited the highest catalytic performance with minimal coke deposition, even during prolonged reaction times. Mechanistic investigations identified oxidative CH_4_ dissociation and efficient electron transfer from Ni to Ce as key factors contributing to the enhanced catalytic activity. This study pioneers the development of defect-engineered fluorite-based catalysts with superior metal–support interactions for efficient DRM applications.

## 2 Materials and Methods

### 2.1 Synthesis of La_2–*x*_Sr_*x*_Ce_2–*y*_Ni_*y*_O_7−δ_ (*x* = 0, 0.1; *y* = 0, 0.1, 0.3, 0.5)

The solution combustion method was used to synthesize a series of oxides: La_2–*x*_Sr_*x*_Ce_2–*y*_Ni_*y*_O_7−δ_ (*x* = 0, 0.1; *y* = 0, 0.1, 0.3, and 0.5). Water-soluble precursor metal nitrates served as the oxidizers, while glycine acted as the fuel. The oxidizer-to-fuel ratio was calculated based on propellant chemistry. For a typical synthesis, the required amounts of La­(NO_3_)_3_·6H_2_O (SRL Chemicals, 99%), Sr­(NO_3_)_2_ (Sigma-Aldrich, 99%), Ce­(NO_3_)_3_·6H_2_O (Sigma-Aldrich, 99%), Ni­(NO_3_)_2_·6H_2_O (SD Fine, 99%), and glycine (C_2_H_5_NO_2_) (Sigma-Aldrich, 99%) were placed in a 300 mL crystallizing dish and dissolved in 45 mL of deionized water. The resulting homogeneous solution was introduced into a muffle furnace preheated to 450 °C. After initial boiling and frothing, the solution underwent combustion, producing flames and yielding a voluminous solid product. The obtained product was cooled to room temperature, ground into a fine powder, and subsequently calcined at 800 °C for 5 h to remove any residual volatile compounds and impurities. The solution combustion method is highly reproducible and easily scalable due to the self-sustaining nature of the combustion process. Furthermore, it is economically viable, employing inexpensive and readily available precursors such as metal nitrates, glycine, and water, without the need for complex reagents or solvents. Compared to conventional coprecipitation or impregnation methods used for supported Ni catalysts, this approach offers significant advantages in terms of synthesis time, cost, and scalability, thereby enhancing the practical applicability of the developed catalyst. For comparison, a 3 wt % Ni–1.65 wt % Sr-doped La_2_Ce_2_O_7_ sample was prepared using the incipient wet impregnation method. Specifically, a stoichiometric amount of synthesized La_2_Ce_2_O_7_ and the required quantities of Ni­(NO_3_)_2_.6H_2_O and Sr­(NO_3_)_2_ were dispersed in minimal amounts of deionized water under continuous stirring. A solution of NaBH_4_ was then added dropwise to the mixture. The suspension was stirred overnight at 80 °C, followed by filtration and drying at 110 °C for 10 h.

### 2.2 Material Characterization

Inductively Coupled Plasma Optical Emission Spectroscopy (ICP-OES) (Agilent Technologies) and energy-dispersive X-ray fluorescence (ED-XRF) spectroscopy (Epsilon 1 PANalytical instrument) were employed to accurately determine the elemental composition and weight ratio of the synthesized catalysts. X-ray diffraction (XRD) analysis was conducted by using a Rigaku Ultima IV diffractometer with Cu Kα radiation (λ = 1.5418 Å) to characterize the structural properties of the catalysts. The measurements were performed at a scan rate of 0.33° min^–1^ with a step size of 0.01°. The microstrain (ε) and the crystallite size (*D*) were determined using the Williamson–Hall Equation, β cosθ = 4ε sinθ + *k*λ/*D*, where β represents the full width at half-maximum, *k* is the shape factor, λ is the characteristic wavelength of Cu Kα radiation, and θ is the corresponding Bragg angle. Additionally, the lattice parameters were determined by refining the experimentally obtained XRD patterns using the Rietveld refinement method with the FullProf-fp2k program. During the atom refinement process within FullProf, the oxygen occupancy was treated as a variable parameter and refined alongside other structural parameters, especially atom refinement. Other parameters include the overall scale factor, background and profile parameters, unit-cell lattice parameters, atomic positions, and thermal parameters. This refinement procedure, utilizing a pseudo-Voigt profile function and linear interpolation between a set of background points as background correction, allowed us to estimate the oxygen occupancy based on the best fit between the calculated and experimental diffraction patterns.

X-ray photoelectron spectroscopy (XPS) analysis was conducted using a Thermo Scientific K-Alpha surface analysis spectrometer with Al K_α_ radiation (1486.6 eV) to investigate the surface elemental bonding and oxidation states. The spectra were calibrated using the C 1*s* peak at 284.8 eV. The structural morphology and surface characterization of the catalysts were examined using field emission scanning electron microscopy (FE-SEM, FEI-Apreo S). High-resolution transmission electron microscopy (HR-TEM) was performed with a JEOL JEM-2100 Plus microscope to determine the catalyst morphology. ImageJ software was used to assess particle diameters and calculate the nickel particle size distribution in the reduced catalyst. EPR analysis was performed using a Bruker ESR 5000. Carbon deposition and mass loss were analyzed by using thermogravimetric analysis (TGA) performed in an oxygen atmosphere with a Shimadzu DTG-60 instrument. The samples were heated from 30 to 900 °C at a ramp rate of 10 °C min^–1^. Confocal Raman spectra were acquired by using a LabRam HR Evolution Raman spectrometer (Horiba-Jobin Yvon) with a laser wavelength of λ = 532 nm and a spectral resolution of 4 cm^–1^. It was checked that the laser heating effect was negligible at the used power (140 μW). H_2_-temperature-programmed reduction (H_2_-TPR) was performed by using a BELCAT-M (BEL Japan) instrument equipped with a thermal conductivity detector (TCD). Typically, 45 mg of catalyst was pretreated with He gas at 150 °C for 1 h. After cooling to 50 °C, a 10% H_2_/He mixture was passed over the sample at a total flow rate of 30 mL min^–1^ for 1 h. The temperature was then ramped from 50 to 900 °C at a rate of 10 °C min^–1^. CO_2_-temperature-programmed desorption (CO_2_-TPD) was also performed using the same equipment. For CO_2_-TPD, 45 mg of catalyst was pretreated in He at 150 °C for 1 h, followed by stabilization at 50 °C for 1 h under a 10% CO_2_/He flow at 30 mL min^–1^. Subsequently, the CO_2_ flow was stopped, and TPD profiles were recorded while ramping the temperature to 750 °C at a rate of 10 °C min^–1^. CO_2_-TPD-MS and CO pulse studies were carried out in an Altamira Instrument (AMI) connected to a thermal conductivity detector (TCD). For TPD-MS, 100 mg of catalyst was pretreated in Ar at 150 °C for 30 min, then cooled to 45 °C. CO_2_ (10% in Ar, 45 mL/min) was adsorbed for 1 h at 45 °C. After stopping the CO_2_ flow, Ar was passed (50 mL/min) from 45 to 800 °C at 10 °C/min. Mass spectra were collected throughout the experiment. Pulse chemisorption studies were conducted to determine the Ni dispersion and the number of active sites. For each experiment, 100 mg of the catalyst was loaded into a U-shaped quartz tube reactor. Prior to the analysis, surface contaminants were removed by degassing the catalyst under a helium flow at 150 °C for 30 min. Subsequently, a gaseous mixture of 5% CO in He was pulsed through the reactor at a flow rate of 60 mL/min. The effluent gas was monitored using a Thermal Conductivity Detector (TCD) to quantify CO adsorption over 20 consecutive pulses. *In situ* Fourier-transform infrared (FTIR) spectroscopy was performed by using a PerkinElmer Spectrum 3 FTIR instrument. The catalyst was placed inside the reaction chamber at elevated temperatures and sealed with a ZnSe-windowed dome. The chamber was equipped with a Praying Mantis attachment and a PID controller (Harrick, USA). The catalyst was heated to 400 °C under a N_2_ atmosphere while recording the background spectrum. Subsequently, reaction gases were introduced, and infrared spectra were collected every 5 min for up to 40 min.

### 2.3 Computational Studies

The density functional theory (DFT) calculations were performed using the projector-augmented-wave (PAW) pseudopotentials with Perdew–Burke–Ernzerhof (PBE) as the exchange-correlation functional with the plane-wave basis set, which has a kinetic energy cutoff of 500 eV as implemented in the Vienna Ab-initio Simulation Package (VASP 6.3.2).^24–27^ The calculations were performed on a pure unit cell (*a* = *b* = *c* = 11.21 Å, α = β = γ = 90°) of La_2_Ce_2_O_7_ containing 88 atoms with a structural composition of (La:Ce:O = 16:16:56). We approximate the composition as La_1.875_Sr_0.125_Ce_1.875_Ni_0.125_O_7_ (instead of La_1.9_Sr_0.1_Ce_1.9_Ni_0.1_O_7_) and similarly for other compositions of La_2–*x*_Sr_*x*_Ce_2–*y*_Ni_*y*_O_7−δ_ (*x* = 0.1; *y* = 0.3, 0.5). Structural modifications were applied to the pure unit cell to form doped systems, followed by geometry and lattice optimization, and spin-dependent total and partial density of states (PDOS) for both pure and doped systems were performed. The *k*-point meshes used were 3 × 3 × 3 for structural optimization and 6 × 6 × 6 for VBM and CBM, DOS calculations. To investigate the valence band maximum (VBM) and conduction band minimum (CBM) in relation to the Fermi level, the charge density distributions of VBM and CBM obtained were visualized using visual molecular dynamics (VMD).^28^

### 2.4 Catalytic Activity Studies

The freshly prepared materials were pelletized and sieved to 60/80 mesh before being placed in a quartz fixed-bed reactor. The catalyst weighed 200 mg, with the bed length maintained at 1 cm. The reactor had an internal diameter of 4 mm and an external diameter of 6 mm. The catalyst was positioned between two ceramic wool plug segments. Subsequently, the quartz reactor was placed in a tube furnace equipped with a temperature controller. The temperature was monitored by using a K-type thermocouple installed within the reactor. The total flow rate of the gas mixture was set at 100 mL min^–1^ (at NTP), corresponding to a gas hourly space velocity (GHSV) of 47,770 h^–1^. The individual flow rates of CH_4_ and CO_2_ were maintained at 10 mL min^–1^, with N_2_ serving as the balance gas. The gas cylinders were procured from CHEMIX Gases Pvt. Ltd., Bangalore. After mixing in a manifold, the gas flow was measured by using a bubble flow meter. The product gases were collected using a 500-μL Hamilton syringe and analyzed with a 5765 Nucon Gas Chromatograph for CO, CH_4_, CO_2_, and H_2_ detection. The analysis was conducted using a Flame Ionization Detector connected to a methanizer along with a Thermal Conductivity Detector.

The percentage conversion is calculated as follows:

5 $$CO_{2}\;\mathrm{conversion}\;\left(\%\right)=\frac{F{[CO_{2}]}_{\mathrm{in}}-F{[CO_{2}]}_{\mathrm{out}}}{F{[CO_{2}]}_{\mathrm{in}}}\times 100$$

6 $$CH_{4}\;\mathrm{conversion}\;\left(\%\right)=\frac{F{[CH_{4}]}_{\mathrm{in}}-F{[CH_{4}]}_{\mathrm{out}}}{F{[CH_{4}]}_{\mathrm{in}}}\times 100$$

where *F*[CO_2_]_in_ are *F*[CH_4_]_in_ are the inlet molar flow rates; *F*[CO_2_]_out_ and *F*[CH_4_]_out_ are the outlet molar flow rates.

Similarly, the product concentration ratio and space-time yield velocity (STY) of each product are calculated as

7 $$\frac{H_{2}}{\mathrm{CO}}\mathrm{ratio}=\frac{\mathrm{moles}\;\mathrm{of}\;H_{2\;}\mathrm{produced}}{\mathrm{moles}\;\mathrm{of CO produced}}$$

8 $$\mathrm{STY of}\;H_{2}=\frac{\mathrm{moles of}\;H_{2\;}\mathrm{produced}}{W\times \mathrm{time}}$$

9 $$\mathrm{STY of CO}=\frac{\mathrm{moles of CO produced}}{W\times \mathrm{time}}$$

The coke formation rate is calculated as

10 $$\mathrm{Coke formation rate}=\frac{\mathrm{Wt.loss}}{W\times \mathrm{time}}$$

where Wt.loss is the weight difference calculated from TGA, *W* is the weight of the catalyst taken, and time is the duration of the reaction.

Turn over frequencies (TOF) for the product gases are calculated as follows

11 $${\mathrm{TOF}}_{H_{2}}=\frac{\mathrm{moles}\;\mathrm{of}\;H_{2\;}\mathrm{produced}}{\mathrm{moles of catalyst used}\times \mathrm{time}}$$

12 $${\mathrm{TOF}}_{\mathrm{CO}}=\frac{\mathrm{moles of CO produced}}{\mathrm{moles of catalyst used}\times \mathrm{time}}$$

## 3 Results and Discussion

### 3.1 Structural Analysis

The pristine La_2_Ce_2_O_7_ and doped materials La_1.9_Sr_0.1_Ce_1.9_Ni_0.1_O_7_, La_1.9_Sr_0.1_Ce_1.7_Ni_0.3_O_7_, and La_1.9_Sr_0.1_Ce_1.5_Ni_0.5_O_7_ were synthesized via a single-step solution combustion method. The combination of a low initiation temperature and the generation of high transient temperatures during the combustion of liquid precursors facilitates the formation of nanocrystalline, highly porous oxides with a large surface area and a uniform distribution of dopants. The synthesized materials were initially evaluated for their elemental composition. Experimental bulk elemental compositions obtained through ICP-OES and XRF characterization were found to closely align with the theoretically calculated values (Table S1). The powder XRD patterns of the pristine and doped oxides are listed in Figure 1a. Detailed analysis and indexing confirmed that the materials crystallized in a defective cubic fluorite structure (*Fm*3̅*m*), with no evidence of the *Fd*3̅*m* pyrochlore phase.^12,29^ The ionic radii of La^3+^ (1.16 Å) and Ce^4+^ (0.87 Å) result in a radius ratio (*R*_A_/*R*_B_) of 1.33, consistent with the defective fluorite structure. Furthermore, no peaks corresponding to individual oxides (e.g., La_2_O_3_, NiO, and SrO) were observed, confirming the formation of a defective fluorite solid solution. However, there could be a possibility of the presence of LaNiO_3_ as a minor impurity in La_1.9_Sr_0.1_Ce_1.5_Ni_0.5_O_7_ (indicated with an asterisk in Figure 1a). The crystallite sizes, calculated using the Williamson–Hall equation based on the (111) plane, were in the range of 9–12 nm for the synthesized materials. Interestingly, the doping of Ni at the B-site caused a shift in the 2θ values toward lower Bragg angles (Figure S1a). The observed decrease in peak intensity and increased broadening in the doped samples are probably due to lattice distortions and defects introduced by the dopant. Since the Shannon radii of the dopants Ni^2+^ (6-fold coordination = 0.69 Å) are smaller than those of the host ions Ce^4+^ (6-fold coordination = 0.87 Å) and La^3+^ (8-fold coordination = 1.16 Å), a dominant contraction effect leading to a decrease in unit cell volume is expected for the doped solid solutions relative to pristine La_2_Ce_2_O_7_.^30,31^ To investigate further, Rietveld refinement was performed on the diffraction patterns of the synthesized materials by using FullProf software (Figure S1b). X-rays are weakly scattered by oxygen due to its low atomic number; however, with good data quality, oxygen occupancy can be estimated with reasonable constraints and modeling. We performed the Rietveld refinement on the diffraction patterns of the synthesized materials using FullProf software by refining the scale factor, zero shift, background, lattice parameters, peak shape, and isotropic thermal parameters. Oxygen occupancy is strongly correlated with thermal parameters and scale factor. After a stable refinement, we considered refining occupancy. The refined parameters, including the unit cell length, cell volume, and oxygen occupancy, are summarized in Table S2. The variation in the unit cell volume with different Ni doping levels is plotted in Figure 1b. Surprisingly, the unit cell volume initially decreased upon Ni doping in La_1.9_Sr_0.1_Ce_1.9_Ni_0.1_O_7_ but increased with higher doping levels in La_1.9_Sr_0.1_Ce_1.7_Ni_0.3_O_7_ and La_1.9_Sr_0.1_Ce_1.5_Ni_0.5_O_7_. The initial decrease can be attributed to the formation of a substitutional solid solution. However, the subsequent increase in cell volume, despite the smaller ionic radii of the dopants, is likely due to the interstitial occupancy of Ni atoms within the host lattice.^32^ To further validate this, energy minimization of the pristine and doped structures was performed by using DFT calculations. These calculations considered Ni substituting Ce in La_1.9_Sr_0.1_Ce_1.9_Ni_0.1_O_7_, as well as Ni occupying interstitial positions alongside Ce substitution in La_1.9_Sr_0.1_Ce_1.7_Ni_0.3_O_7_ and La_1.9_Sr_0.1_Ce_1.5_Ni_0.5_O_7_. For computational efficiency, La_1.875_Sr_0.125_Ce_1.875_Ni_0.125_O_7_ was used as an approximate representation of La_1.9_Sr_0.1_Ce_1.9_Ni_0.1_O_7_, with similar approximations applied for catalysts with higher Ni content. Interestingly, the theoretically calculated energy-minimized super cell volumes for different Ni doping levels, as plotted in Figure 1b, exhibit the same trend as the experimentally obtained unit cell volume. This interstitial incorporation would also significantly increase the strain in the crystal lattice. The strain, calculated using the Williamson–Hall method and plotted in Figure 1b, indeed showed a clear increase in the tensile strain due to the interstitial occupancy of Ni. Microstrain arises from deviations of atoms from their lattice positions and is known to influence catalytic activity.^7,33^

**Figure 1:**
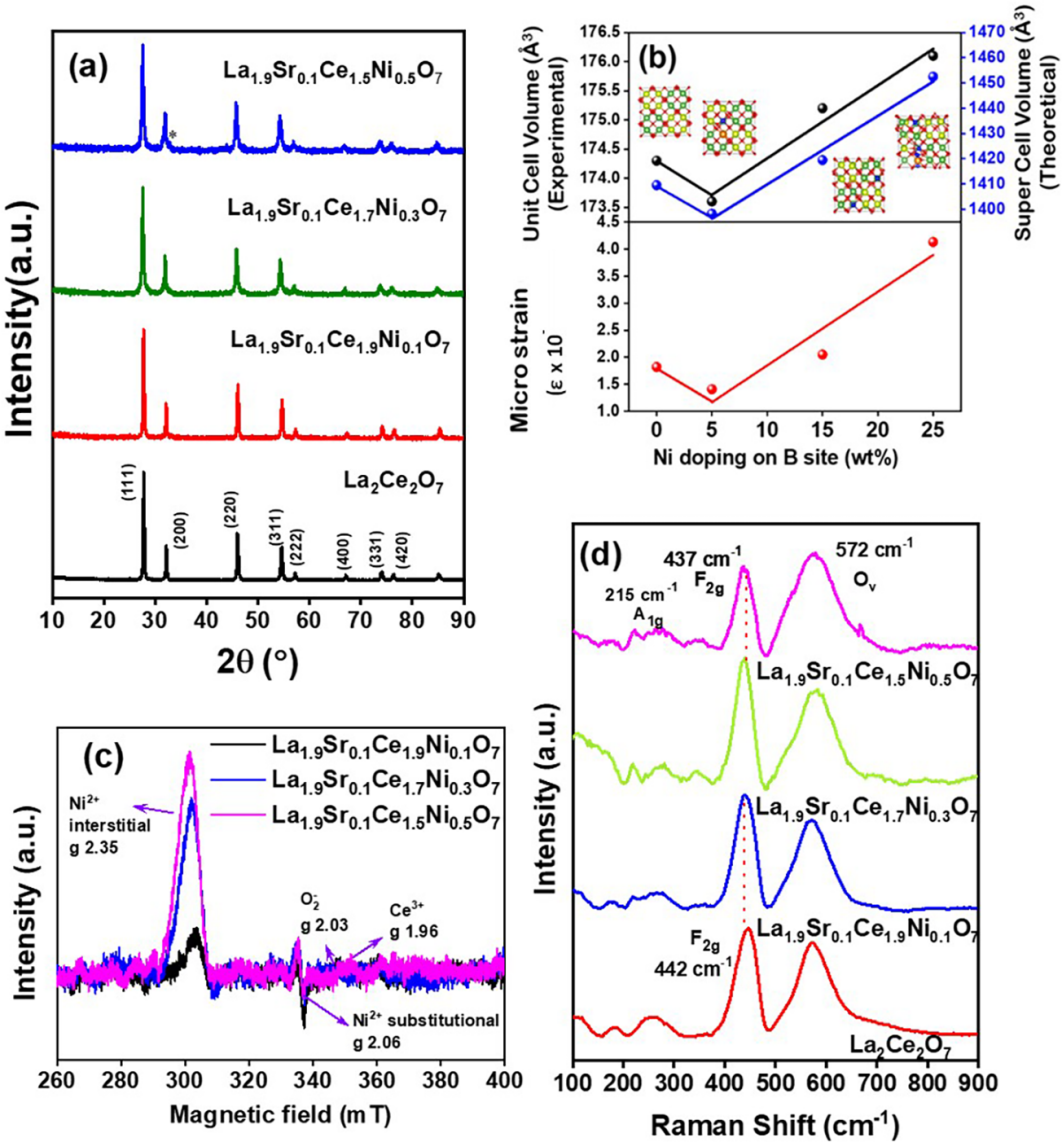
(a) Powder X-ray diffraction patterns of the pristine and doped defective fluorite oxides, (b) variation of unit cell volume and lattice strain with doping of Ni derived from Rietveld refinement, DFT calculations, and the Williamson–Hall method, (c) EPR, and (d) Raman spectra of the synthesized materials.

The presence of Ni^2+^ ions in both the interstitial octahedral voids and substitutional 4*a* sites within the FCC lattice was further confirmed by the EPR spectra shown in Figure 1c. Notably, two distinct Ni^2+^ EPR peaks are observed: one at 337 mT and another in the 310–303 mT region correspond to magnetically inequivalent Ni environments. The peak at the higher magnetic field is attributed to substitutional Ni^2+^ ions (Ni_sub_), whereas the peak at the lower magnetic field likely originates from the interstitial Ni^2+^ ions (Ni_in_).^34–38^ The intensity of the peak associated with Ni_in_ increased with higher Ni^2+^ concentrations, and this peak broadened and shifted to lower magnetic fields (from 303 mT to 301 mT) as doping levels increased.^34,39^ The peak broadening is attributed to dipolar interactions among the multiple magnetic domains—specifically, the interstitial and substitutional domains. The shift to a lower magnetic field is likely due to spin disorder, which arises from enhanced interactions between neighboring spins, inducing antiferromagnetic behavior in the catalyst. Raman spectra of the freshly synthesized pristine and doped catalysts were recorded to provide deeper insights into the lattice structure and to evaluate the presence of oxygen vacancies (Figure 1d). In pristine La_2_Ce_2_O_7_, the band at 442 cm^–1^ corresponds to the Raman-active F_2_g mode arising from the symmetric vibration of eight oxygen atoms surrounding the Ce^4+^/La^3+^ cations in the defective cubic fluorite structure. Upon Ni doping, this band shifts to a lower wavenumber (437 cm^–1^), indicating lattice expansion due to strain-induced relaxation, consistent with diffraction studies.^40,41^ Additionally, the broad band observed at 572 cm^–1^ in the synthesized materials corresponds to both intrinsic and extrinsic oxygen vacancies in the lattice along with the potential Frenkel-type oxygen defects.^40,42,43^ The presence of the weak peak at around 215 cm^–1^ may correspond to the minor phases of the LaNiO_3_ Raman A_1_g band.^12^

The irregular morphology of the pristine and doped oxides was obtained from the FE-SEM images (Figure S2). Figure 2a displays HR-TEM micrographs of pristine La_2_Ce_2_O_7_ nanoparticles with an average size of about 25 nm. The interplanar distances of (111) and (200) planes of La_2_Ce_2_O_7_ (Figure 2b) corroborate the X-ray diffraction result. The observed increase of (111) and (200) interplanar distances in La_2_Ce_2_O_7_ compared to those in CeO_2_ is attributed to the incorporation of larger La^3+^ ions into the lattice. The (111) interplanar distances of La_2_Ce_2_O_7_ and La_1.9_Sr_0.1_Ce_1.9_Ni_0.1_O_7_ were 0.321 and 0.320 nm, respectively, whereas the (200) interplanar distances of La_2_Ce_2_O_7_ and La_1.9_Sr_0.1_Ce_1.9_Ni_0.1_O_7_ were 0.264 and 0.261 nm, respectively (Figure 2b,d). The subsequent decrease of *d*_(111)_ and *d*_(200)_ in La_1.9_Sr_0.1_Ce_1.9_Ni_0.1_O_7_ than that of La_2_Ce_2_O_7_ is due to the substitution of smaller Ni^2+^ in *4a* sites. This shrinkage aligns well with the lattice parameter values observed from Rietveld refinement and DFT studies (*vide* Figure 1b). There was no significant change in the particle morphology (Figure 2c,e,g) and size in the doped solid solutions compared to the pristine La_2_Ce_2_O_7_. The (111) interplanar distances of La_1.9_Sr_0.1_Ce_1.7_Ni_0.3_O_7_ and La_1.9_Sr_0.1_Ce_1.5_Ni_0.5_O_7_ were observed to be 0.322 and 0.324 nm, respectively, whereas the (200) interplanar spacings of La_1.9_Sr_0.1_Ce_1.7_Ni_0.3_O_7_ and La_1.9_Sr_0.1_Ce_1.5_Ni_0.5_O_7_ were 0.283 and 0.300 nm, respectively (Figure 2f,h). The subsequent increase in (111) and (200) interplanar distances despite the smaller ionic radii of the dopants is likely due to the interstitial occupancy of Ni atoms within the host lattice. The change in the *d* spacing is quite in accordance with the data obtained from X-ray diffraction studies. The SAED patterns of the pristine and doped oxides, plotted in Figure S3a–d, demonstrate the polycrystalline nature of the materials. The elemental mapping in Figure S4 reveals a highly uniform dispersion of the dopants across the material, with only minor nanoscale variations in Ni distribution. Negligible Ni–La segregation might be due to the minuscule formation of LaNiO_3_.

**Figure 2:**
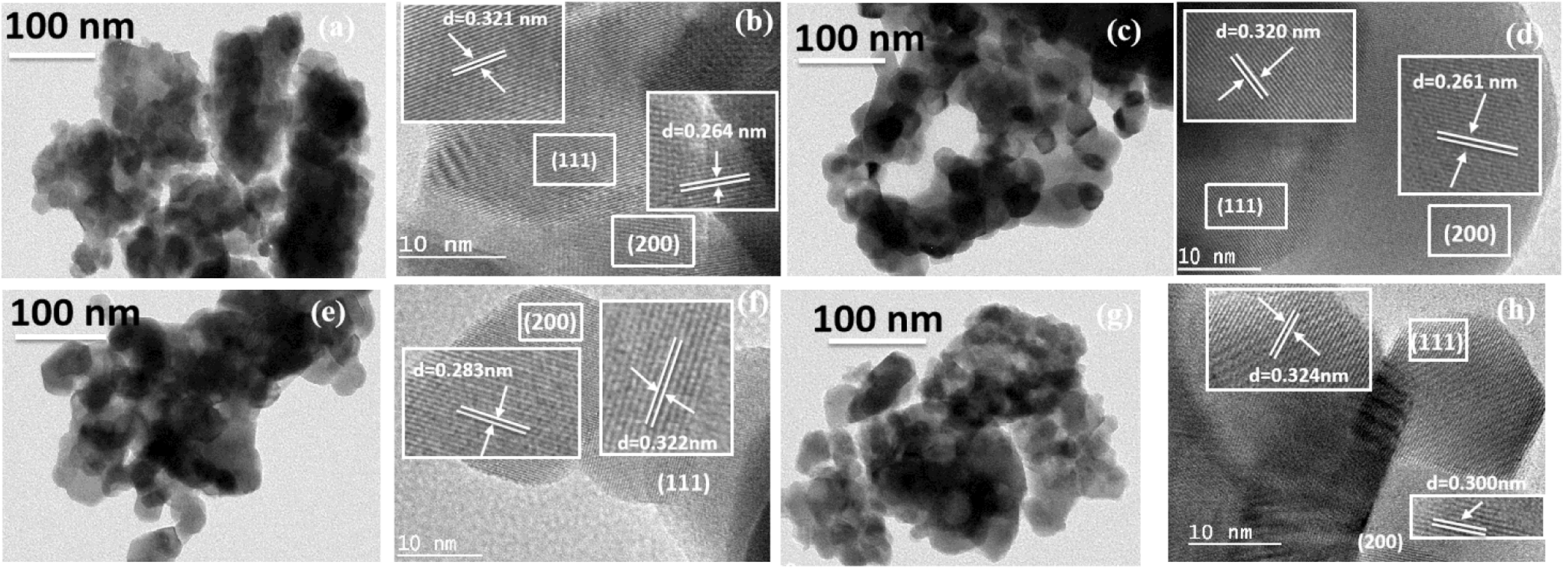
HR-TEM images of (a,b) La_2_Ce_2_O_7_, (c,d) La_1.9_Sr_0.1_Ce_1.9_Ni_0.1_O_7_, (e,f) La_1.9_Sr_0.1_Ce_1.7_Ni_0.3_O_7_, and (g,h) La_1.9_Sr_0.1_Ce_1.5_Ni_0.5_O_7_.

### 3.2 DRM Activity Evaluation

Dry reforming experiments were conducted using freshly synthesized oxides under steady-state conditions for up to 5 h at three temperatures of 650, 700, and 750 °C with purging with 10 vol % CO_2_ and CH_4_. The conversions of the reactants and the H_2_/CO ratio along with the thermodynamic equilibrium are presented in Figure 3a–c. The pristine La_2_Ce_2_O_7_ exhibited very low conversions for both CO_2_ and CH_4_, with minimal production of H_2_ relative to CO indicating negligible DRM activity in the absence of the active Ni metal. Upon the introduction of nickel, La_1.9_Sr_0.1_Ce_1.9_Ni_0.1_O_7_ showed an improvement in the conversion efficiencies for both reactants, achieving an H_2_/CO ratio of ∼0.3. The result highlights the critical role of Ni as an active site in catalyzing the reforming reaction. However, while Ni initiates the DRM reaction, the product distribution is skewed, possibly due to a lower activity toward hydrogen production or the prevalence of side reactions like the RWGS. With further increasing the nickel content, La_1.9_Sr_0.1_Ce_1.7_Ni_0.3_O_7_ displayed significant conversion efficiencies for CO_2_ and CH_4_, with a steady increase in feed reactant conversion as the temperature increased. However, the H_2_/CO ratio remained nearly constant across the temperature range, with a value of 0.8. La_1.9_Sr_0.1_Ce_1.5_Ni_0.5_O_7_ demonstrated 58% CO_2_ conversion and 42% CH_4_ conversion at 650 °C. At higher temperatures of 700 and 750 °C, both CO_2_ and CH_4_ conversions reached approximately 90%, with the H_2_/CO ratio remaining constant. The conversion of the feed reactants approached thermodynamic equilibrium over La_1.9_Sr_0.1_Ce_1.5_Ni_0.5_O_7_ at elevated temperatures. The catalytic activity normalized with respect to the Ni content is shown in Figure S5. It should be noted that, unlike La_1.9_Sr_0.1_Ce_1.9_Ni_0.1_O_7_, the higher Ni-doped solid solutions, La_1.9_Sr_0.1_Ce_1.7_Ni_0.3_O_7_ and La_1.9_Sr_0.1_Ce_1.5_Ni_0.5_O_7_ exhibited the presence of Ni ions within the lattice and interstitial sites, along with lattice strain in the materials. This combination is believed to contribute intricately to the enhanced reforming activity observed in these solid solutions.

**Figure 3:**
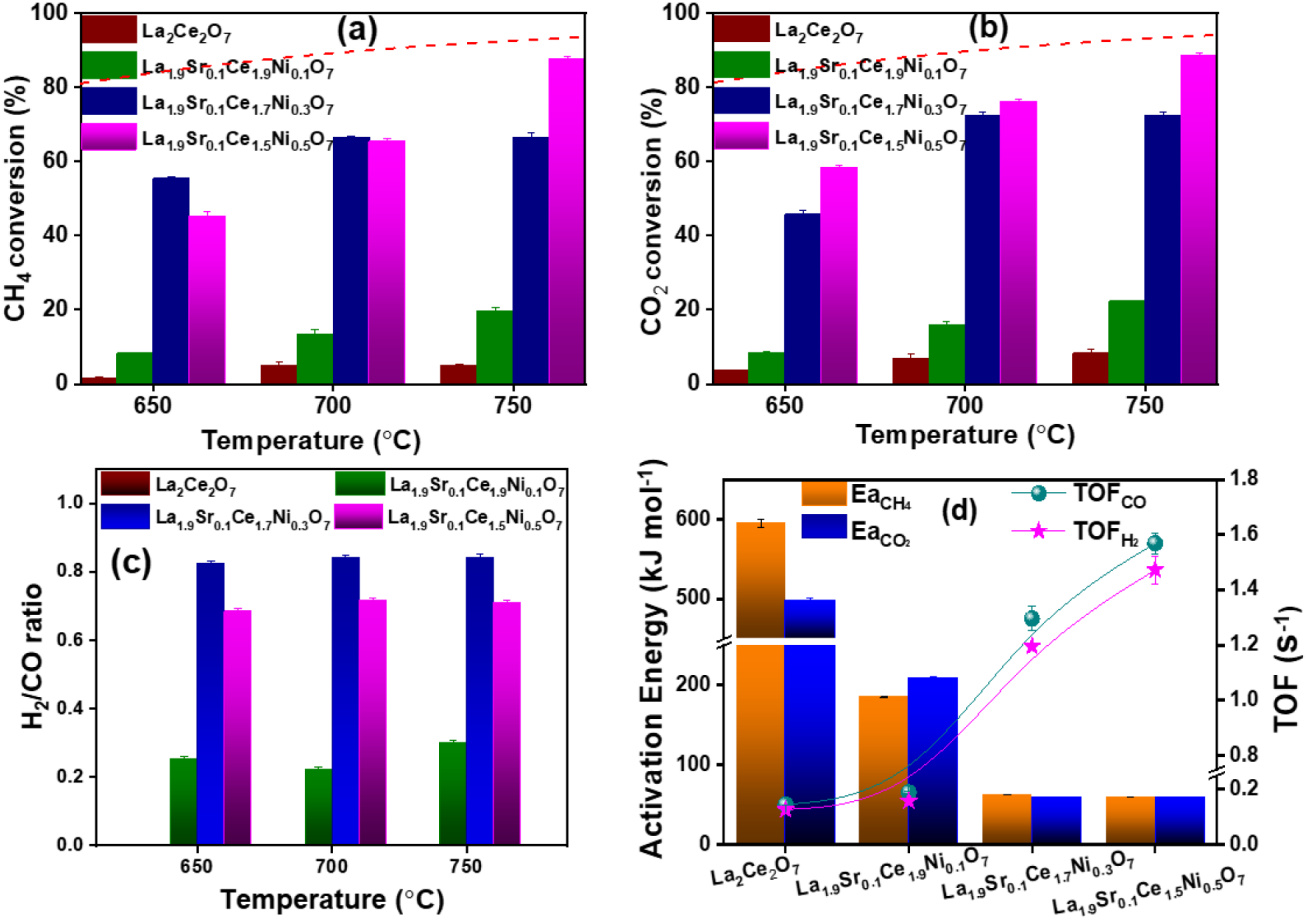
DRM conversion of (a) CH_4_, (b) CO_2_, and (c) H_2_/CO ratio at 650–750 °C over La_2_Ce_2_O_7_, La_1.9_Sr_0.1_Ce_1.9_Ni_0.1_O_7_, La_1.9_Sr_0.1_Ce_1.7_Ni_0.3_O_7_, and La_1.9_Sr_0.1_Ce_1.5_Ni_0.5_O_7_. (d) Activation energies of CO_2_, CH_4_ and TOF of H_2_, and CO at 750 °C. The dashed lines in (a) and (b) represent the thermodynamic equilibrium conversion of CH_4_ and CO_2_.

This intrigued us to evaluate the activation energy of CH_4_ and CO_2_ during the reforming reaction by varying the loading of the solid solution catalysts from 50 to 200 mg using a feed mixture of CH_4_:CO_2_:N_2_ = 10:10:80 vol %. The impact of temperature on the reaction rate was examined in the range of 650–750 °C. A differential flow reactor assumption was followed for the packed bed reactor. A differential flow reactor is assumed to have negligible resistance toward mass transfer and heat transfer. To validate the kinetic regime, experimental conversions of CO_2_ and CH_4_ were plotted as a function of catalyst loading (Figure S6), and the rate of the reaction was calculated as follows:

13 $$-r_{CO_{2}}=\frac{X_{CO_{2}}}{W/F_{CO_{2}}}$$

14 $$-r_{CH_{4}}=\frac{X_{CH_{4}}}{W/F_{CH_{4}}}$$

$-r_{{\mathrm{CO}}_{2}}$ = observed reaction rate, μmol g^–1^ s^–1^, $X_{{\mathrm{CO}}_{2}/{\mathrm{CH}}_{4}}$ = fractional conversion of CO_2_/CH_4_, *W* = catalyst loading, g, $F_{{\mathrm{CO}}_{2}/{\mathrm{CH}}_{4}}$ = molar flow rate, μmol s^–1^. In Figure S6, the fractional conversion of $F_{{\mathrm{CO}}_{2}/{\mathrm{CH}}_{4}}$ varies linearly with the catalyst loading *W* in the reactor. The reaction rates for each catalyst were determined from the slope of $X_{{\mathrm{CO}}_{2}}$ vs ${W/F}_{{\mathrm{CO}}_{2}}$ and $X_{{\mathrm{CH}}_{4}}$ vs ${W/F}_{{\mathrm{CH}}_{4}}$ at 675, 700, 725, and 750 °C. The Mears–Anderson criterion was employed to assess heat and mass transfer to know intraparticle heat transfer transportations, and the Weisz–Prater criterion was employed to evaluate pore diffusion limitations.^44,45^ For La_1.9_Sr_0.1_Ce_1.7_Ni_0.3_O_7_, the deviations from linearity and the potential plateauing observed at higher temperatures (especially at 725 and 750 °C) suggest that diffusion limitations might be playing a role. As the intrinsic reaction rate increases with temperature, the rate of reactant transport to the active sites may become a limiting factor.^46^ The apparent activation energies were calculated from the Arrhenius plots (inset of Figure S6) and are presented in Figure 3d. The apparent activation energies for CO_2_ were 594.7 kJ mol^–1^ and those for CH_4_ were 498.3 kJ mol^–1^ over La_2_Ce_2_O_7_. The activation energies for CH_4_ and CO_2_ were 185.5 and 209.0 kJ mol^–1^, respectively, over La_1.9_Sr_0.1_Ce_1.9_Ni_0.1_O_7_. These values significantly decreased to 62.5 and 59.3 kJ mol^–1^, respectively, over La_1.9_Sr_0.1_Ce_1.7_Ni_0.3_O_7_. Similar activation energies were also observed with La_1.9_Sr_0.1_Ce_1.5_Ni_0.5_O_7_. A significant reduction in the activation energies for CH_4_ and CO_2_ was observed when Ni ions were incorporated into the lattice and interstitial positions of La_1.9_Sr_0.1_Ce_1.7_Ni_0.3_O_7_ and La_1.9_Sr_0.1_Ce_1_._5_Ni_0_._5_O_7_. The TOF values were calculated using eqs 11 and 12 and are presented in Figure 3d. For pristine La_2_Ce_2_O_7_, the TOF value for CO was only 0.03 s^–1^. Upon substitution with Ni in La_1.9_Sr_0.1_Ce_1.9_Ni_0.1_O_7_, it slightly increased to 0.16 s^–1^ for CO and 0.05 s^–1^ for H_2_. Further Ni incorporation led to a significant enhancement in the TOF, reaching 1.1 s^–1^ for CO and 0.9 s^–1^ for H_2_ in La_1.9_Sr_0.1_Ce_1.7_Ni_0.3_O_7_. The pronounced reduction in activation energies upon interstitial Ni incorporation can be attributed to enhanced lattice oxygen mobility and the formation of additional oxygen vacancies, which facilitate CO_2_ activation and CH_4_ dissociation. Moreover, the introduction of Ni into interstitial sites modifies the local electronic structure of the La–Ce–O lattice, which might enhance the activation of CO_2_ and CH_4_. These synergistic effects collectively lower the energy barriers for surface reactions, thereby enhancing the overall catalytic activity. The maximum TOF values were obtained for La_1.9_Sr_0.1_Ce_1.5_Ni_0.5_O_7_ with 1.5 s^–1^ for CO and 1.3 s^–1^ for H_2_. Comprehensive studies clearly indicate that the Ni ions within the lattice and interstitial sites, coupled with the lattice strain in the materials, played a pivotal role in enhancing the reforming activity by lowering the activation energies and increasing the TOF.

To validate the importance of ionic Ni within the lattice and interstitial positions, a reference catalyst, 1.6% Sr/3.3% Ni/La_2_Ce_2_O_7_, was synthesized by impregnating Sr and Ni onto the surface of La_2_Ce_2_O_7_. The amounts of Sr and Ni in this catalyst matched those in La_1.9_Sr_0.1_Ce_1.7_Ni_0.3_O_7_. The comparative conversion data of the feed gases are plotted in Figure 4a,b. Interestingly, the impregnated catalyst exhibited significantly lower DRM activity compared with the solid solution catalysts. These results conclusively demonstrate that Ni ions, located in both the lattice and interstitial positions of the pyrochlore structure, serve as pivotal active sites for C–H bond activation in CH_4_, offering superior performance compared to Ni dispersed on the surface. The ionic Ni and Sr in the solid solutions also contribute to creating oxygen vacancies, as confirmed by Raman studies. These vacancies facilitate CO_2_ activation through C–O bond dissociation. To further investigate the role of oxygen vacancies formed due to Sr at the A-site, a Sr-free catalyst, La_2_Ce_1.7_Ni_0.3_O_7_, was prepared, and its DRM activity was assessed as a control experiment. While the CO_2_ and CH_4_ conversions over La_2_Ce_1.7_Ni_0.3_O_7_ were comparable to those of La_1.9_Sr_0.1_Ce_1.7_Ni_0.3_O_7_, the formation of syngas was insignificant, with a H_2_/CO ratio of ∼0.4 (Figure 4c). Figure 4d shows the STY of H_2_ and CO for 1.6% Sr/3.3% Ni/La_2_Ce_2_O_7_, La_2_Ce_1.7_Ni_0.3_O_7_, and La_1.9_Sr_0.1_Ce_1.7_Ni_0.3_O_7_. The STY was lowest for 1.6% Sr/3.3% Ni/La_2_Ce_2_O_7_ and highest for La_1.9_Sr_0.1_Ce_1.7_Ni_0.3_O_7_. The low ratio may be attributed to significant unselective reverse water–gas shift and/or CO_2_ methanation reactions over La_2_Ce_1.7_Ni_0.3_O_7_. The controlled experiments emphasize the collective importance of oxygen vacancies introduced by Sr at the A-site for efficient C–O bond dissociation in CO_2_, as well as the strategic placement of Ni ions at the B-site and interstitial positions for effective C–H bond activation in CH_4_ to selectively produce syngas. The XRD patterns and the FE-SEM images of the exhausted catalysts provided in Figure S7a do not show any changes in crystallinity or morphology from the as-prepared catalysts.

**Figure 4:**
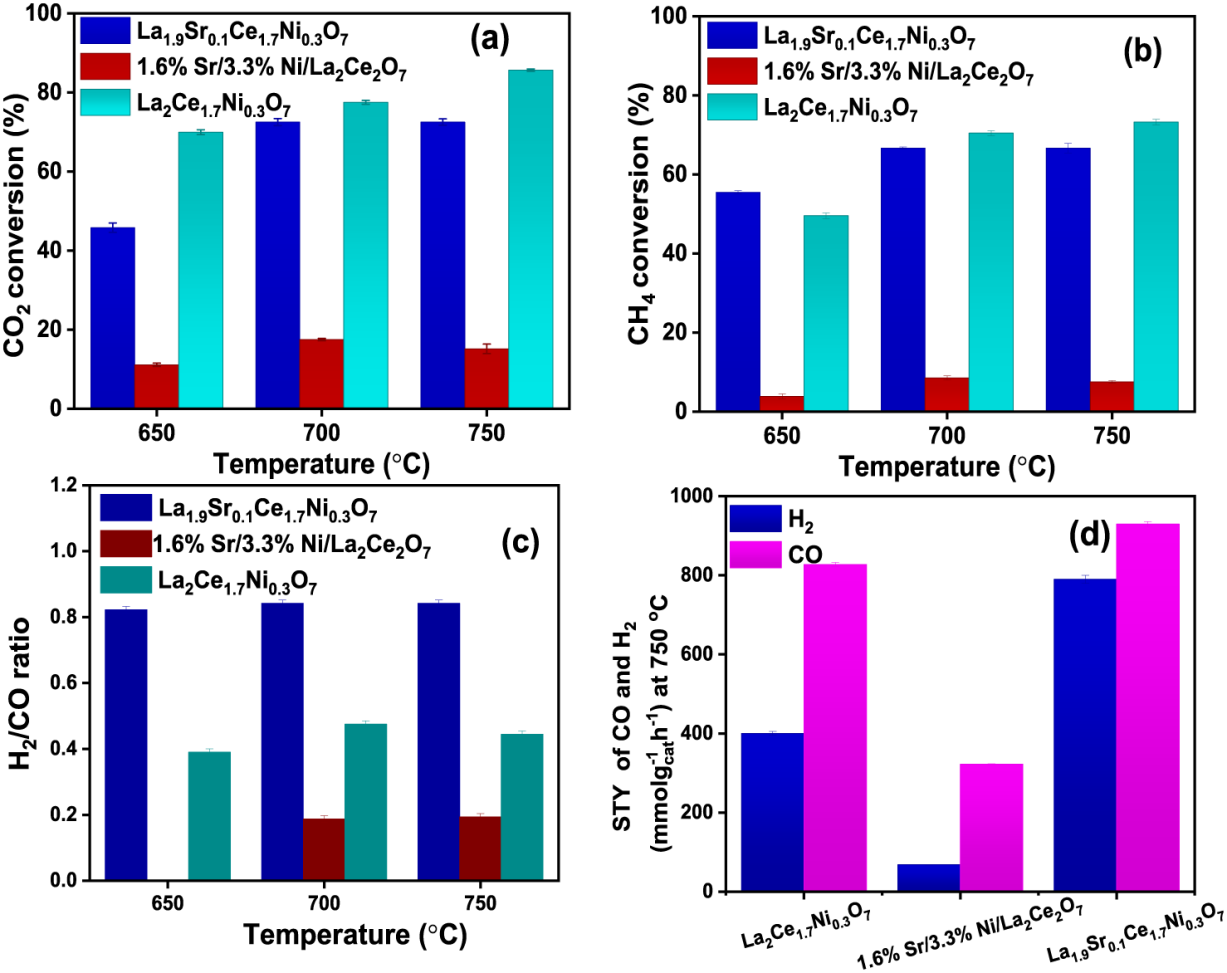
Conversion of (a) CH_4_, and (b) CO_2_ with the (c) H_2_/CO ratio during DRM at the temperatures of 650, 700, and 750 °C over 1.6% Sr/3.3% Ni/La_2_Ce_2_O_7_, La_1.9_Sr_0.1_Ce_1.7_Ni_0.3_O_7_, and La_2_Ce_1.7_Ni_0.3_O_7_. (d) STY of H_2_ and CO over 1.6% Sr/3.3% Ni/La_2_Ce_2_O_7_, La_1.9_Sr_0.1_Ce_1.7_Ni_0.3_O_7_, and La_2_Ce_1_._7_Ni_0_._3_O_7_ at 700 °C.

Both the highly active catalysts La_1.9_Sr_0.1_Ce_1.7_Ni_0.3_O_7_ and La_1.9_Sr_0.1_Ce_1.5_Ni_0_._5_O_7_ were studied for 50 h of catalytic performance and stability at the optimum temperature of 700 °C, and the results are reported in Figure 5a,b. Both materials demonstrated robust stable conversion of feed reactants for up to 50 h. La_1.9_Sr_0.1_Ce_1.7_Ni_0.3_O_7_ showed ∼70% conversion of CO_2_ and CH_4_ with an H_2_/CO ratio of 0.7, whereas La_1.9_Sr_0.1_Ce_1.5_Ni_0.5_O_7_ exhibited mildly superior catalytic activity with more than 85% conversion of CH_4_ and CO_2_, and H_2_/CO ratio of 0.8. Thermogravimetric studies were performed over both the exhausted catalysts after 50 h of time-on-stream experiments to evaluate the quantity of coke formation from methane decomposition or the Boudouard reaction, and the average rate of coke formation is shown in Figure 5c. Apparently, the average coke formation over La_1.9_Sr_0.1_Ce_1.7_Ni_0.3_O_7_ and La_1.9_Sr_0.1_Ce_1.5_Ni_0.5_O_7_ was found to be as low as 11 and 12 μg_c_ g_cat_^–1^ h^–1^, respectively, after 50 h of time-on-stream experiments. A comparison of the weight loss of the exhausted catalysts reported in the literature (Table S3). and the catalytic performance of various DRM systems highlights the superior coke resistance and high activity of the present La_1.9_Sr_0.1_Ce_1.5_Ni_0.5_O_7_ catalyst under relatively mild conditions. Among the benchmark catalysts, Ni-based systems such as Pt/Mg_1_–_*x*_Ni_*x*_O, core–shell Ni@Al_2_O_3_, and MgO–Al_2_O_3_ composites exhibit CH_4_ and CO_2_ conversions in the range of 80–95%, but typically require higher temperatures (750–900 °C) and suffer from considerable carbon deposition (6–30%), leading to gradual deactivation. Noble metal catalysts such as Rh/ZrO_2_ show good stability with minimal coke formation due to the rapid oxidation of CH_*x*_ intermediates; however, their activity remains moderate (CH_4_: 53%, CO_2_: 70%), and the high cost limits large-scale applicability. In contrast, the La_1.9_Sr_0.1_Ce_1.5_Ni_0.5_O_7_ catalyst achieves 83.5% CH_4_ conversion and 86% CO_2_ conversion at only 700 °C, with a low coke deposition of 2.2%, outperforming most conventional Ni-based systems and approaching the stability of Rh-based catalysts. The improved performance can be attributed to the enhanced oxygen mobility and synergistic interaction between Ce^4+^/Ce^3+^ redox pairs and Ni active sites, which facilitate CO_2_ activation and suppress carbon accumulation.

**Figure 5:**
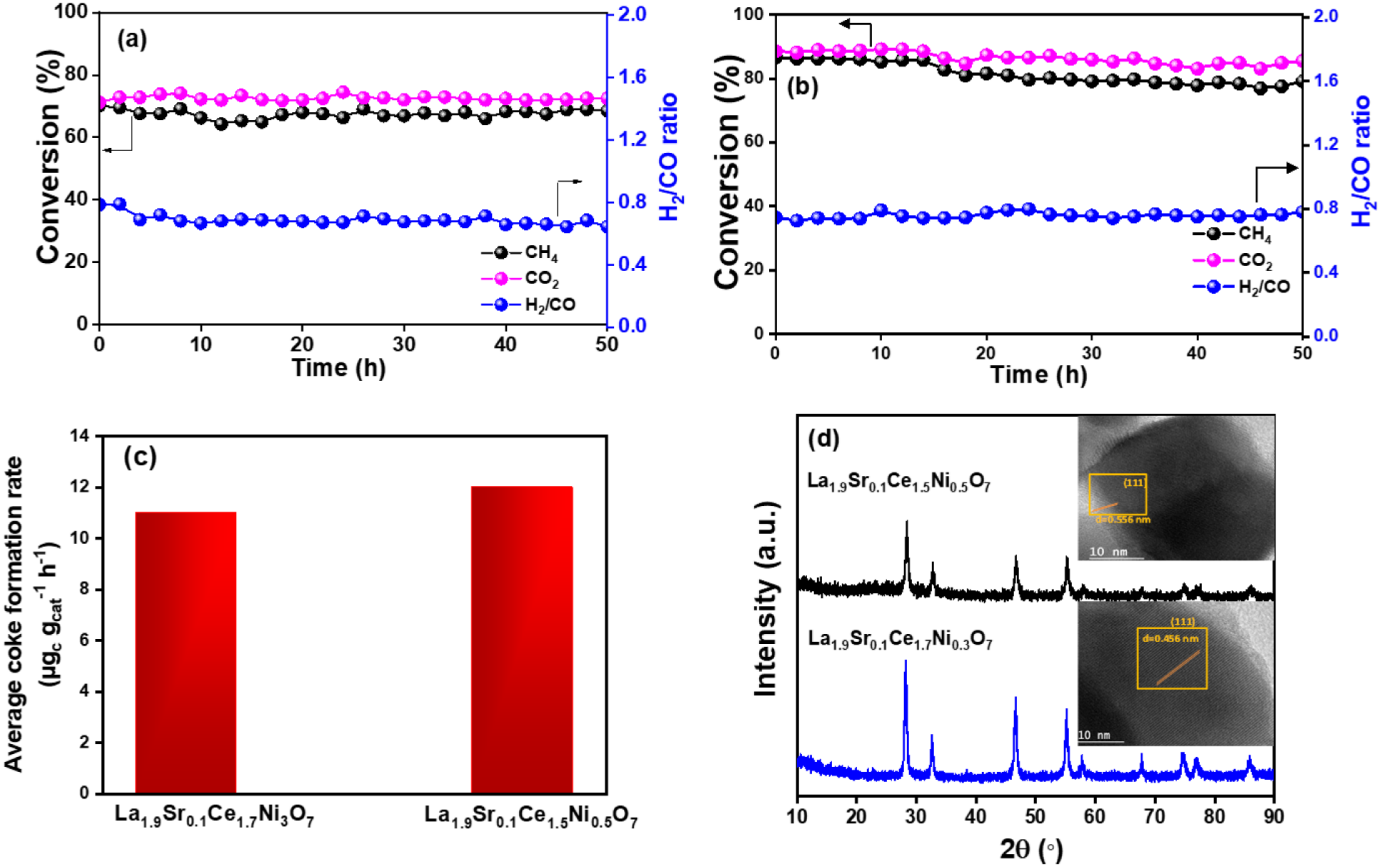
Reactant conversion and H_2_/CO ratio with 50-h time on stream stability studies for (a) La_1.9_Sr_0.1_Ce_1.7_Ni_0.3_O_7_ and (b) La_1.9_Sr_0.1_Ce_1.5_Ni_0.5_O_7_ at 700 °C. (c) Coke formation rate for La_1.9_Sr_0.1_Ce_1.7_Ni_0.3_O_7_ and La_1.9_Sr_0.1_Ce_1.5_Ni_0.5_O_7_. (d) Powder XRD and HR-TEM of the 50 h exhausted a_1.9_Sr_0.1_Ce_1.7_Ni_0.3_O_7_ and La_1.9_Sr_0.1_Ce_1.5_Ni_0.5_O_7_.

The XRD profile of the two exhausted catalysts after 50 h of reaction is provided in Figure 5d. The exhausted catalysts showed similar XRD patterns as those of the as-prepared materials, indicating the robust structural stability of the defect fluorite catalysts. Further, the Rietveld refinement of the 50 h exhausted La_1.9_Sr_0.1_Ce_1.7_Ni_0.3_O_7_ (Figure S7b, χ^2^ = 2.52) also exhibited a similar lattice parameter (5.59 Å) and cell volume 175.58 Å^3^ as the fresh La_1.9_Sr_0.1_Ce_1.7_Ni_0.3_O_7_ (*vide* Table S2). The refinement corroborates the structural integrity of the catalysts. The HR-TEM micrographs in the inset of Figure 5d show no prominent coke deposits on the exhausted catalysts. The elemental mapping of the exhausted catalyst in Figure S7c reveals the retention of the highly uniform dispersion of the dopants across the material. Minor nanoscale variations of Ni distribution and negligible Ni–La segregation were also observed as the fresh catalysts.

### 3.3 Catalytic Mechanism and Metal–Support Interaction

The superior catalytic activity with remarkable stability and negligible coke formation intrigued us to understand the reaction mechanism over the Ni-doped defect fluorite catalysts. Thus, H_2_-TPR experiments were carried out to study the reducibility of the materials (Figure S8a). One can observe that the reducibility significantly increased with the introduction of Ni and apparently doubled with higher doping of Ni, as can be observed from the shifting of the Ce and Ni reduction peaks toward lower temperatures. The amount of H_2_ consumed was found to be 164.8, 305.6, 694.8, and 660.4 μmol/g, respectively, for La_2_Ce_2_O_7_, La_1.9_Sr_0.1_Ce_1.9_Ni_0.1_O_7_, La_1.9_Sr_0.1_Ce_1.7_Ni_0.3_O_7_, and La_1.9_Sr_0.1_Ce_1.5_Ni_0.5_O_7._ For undoped La_2_Ce_2_O_7_, a single broad reduction peak around 587 °C corresponds to the reduction of surface Ce^4+^ species. Ni doping significantly enhances the reducibility: La_1.9_Sr_0.1_Ce_1.9_Ni_0.1_O_7_ exhibits multiple reduction events, including the low-temperature reduction of Ni^2+^ and the high-temperature reduction of Ce^4+^ to Ce^3+^.^47^ Increasing the Ni content further lowers the reduction temperatures due to Ni-mediated H_2_ spillover and leads to enhanced H_2_ uptake in La_1.9_Sr_0.1_Ce_1.7_Ni_0.3_O_7_. However, at higher Ni loading, as in La_1.9_Sr_0.1_Ce_1.5_Ni_0.5_O_7_, the H_2_ consumption does not increase linearly. The Ni in the interstitial position significantly enhanced the reducibility of Ce, which could be due to the metal–support interaction and the H-spillover phenomenon in the materials.^48,49^ This redox flexibility directly correlates with the catalyst’s outstanding dry reforming activity and resistance to coke formation.

The basicity of the materials was investigated by CO_2_-TPD studies, and the corresponding desorption profiles are shown in Figure S8b. Peaks around 100 °C most likely represent weak basic sites, whereas peaks around 240 °C and above 500 °C represent medium and strong basic sites, respectively. These stronger sites promote CO_2_ adsorption and activate it more effectively, enhancing its reactivity with methane-derived species.^50^ Apparently, La_1.9_Sr_0.1_Ce_1.7_Ni_0.3_O_7_ and La_1.9_Sr_0.1_Ce_1.5_Ni_0.5_O_7_ possess the maximum number of medium and strong basic sites, which can be crucial for higher DRM activity (Table S4). The augmentation of strong basic sites is a direct consequence of enhanced oxygen vacancy formation, demonstrating a positive correlation with CO_2_ activation. The significant desorption of CO_2_ from the medium and strong basic sites of La_1.9_Sr_0.1_Ce_1.7_Ni_0.3_O_7_ has been corroborated by the CO_2_ TPD-MS signal as well (Figure S8c).

Detailed XPS analyses of both freshly synthesized and exhausted catalysts were conducted to evaluate the variations in the electronic interactions during DRM activity. The survey spectra of the freshly synthesized catalysts are given in Figure S9, and the C 1*s* spectra of the freshly synthesized and exhausted catalysts are plotted in Figure S10. The core-level spectra of the overlapped region of La 3*d* and Ni 2*p* from freshly prepared catalysts and those subjected to 15 h of time-on-stream experiments are presented in Figure 6a and b, respectively. The Ni 2*p* region exhibited Ni 2*p*_3/2_ and 2*p*_1/2_ peaks corresponding to Ni^2+^ and Ni^3+^ ions. The Ni^3+^ peak appeared at a higher binding energy (2*p*_3/2_–2*p*_1/2_ at 856.3–858.2 eV) compared to Ni^2+^ (2*p*_3/2_–3*p*_1/2_ at 853.7–855.5 eV).^51,52^ The deconvolution of La 3*d* was not carried out for brevity. Interestingly, after time-on-stream experiments, the oxidation of Ni^2+^ to Ni^3+^ was detected after DRM in the catalysts. To delve deeper, we also analyzed the Ni 3*p* region, which corroborated the oxidation of Ni^2+^ to Ni^3+^ after the DRM reaction (Figure S11). In the reducing environment of DRM, the oxidation of active-site Ni^2+^ must be accompanied by a parallel reduction reaction. This parallel reduction reaction could be the reduction of Ce^4+^ → Ce^3+^ owing to the highly reducible nature of the support.

**Figure 6:**
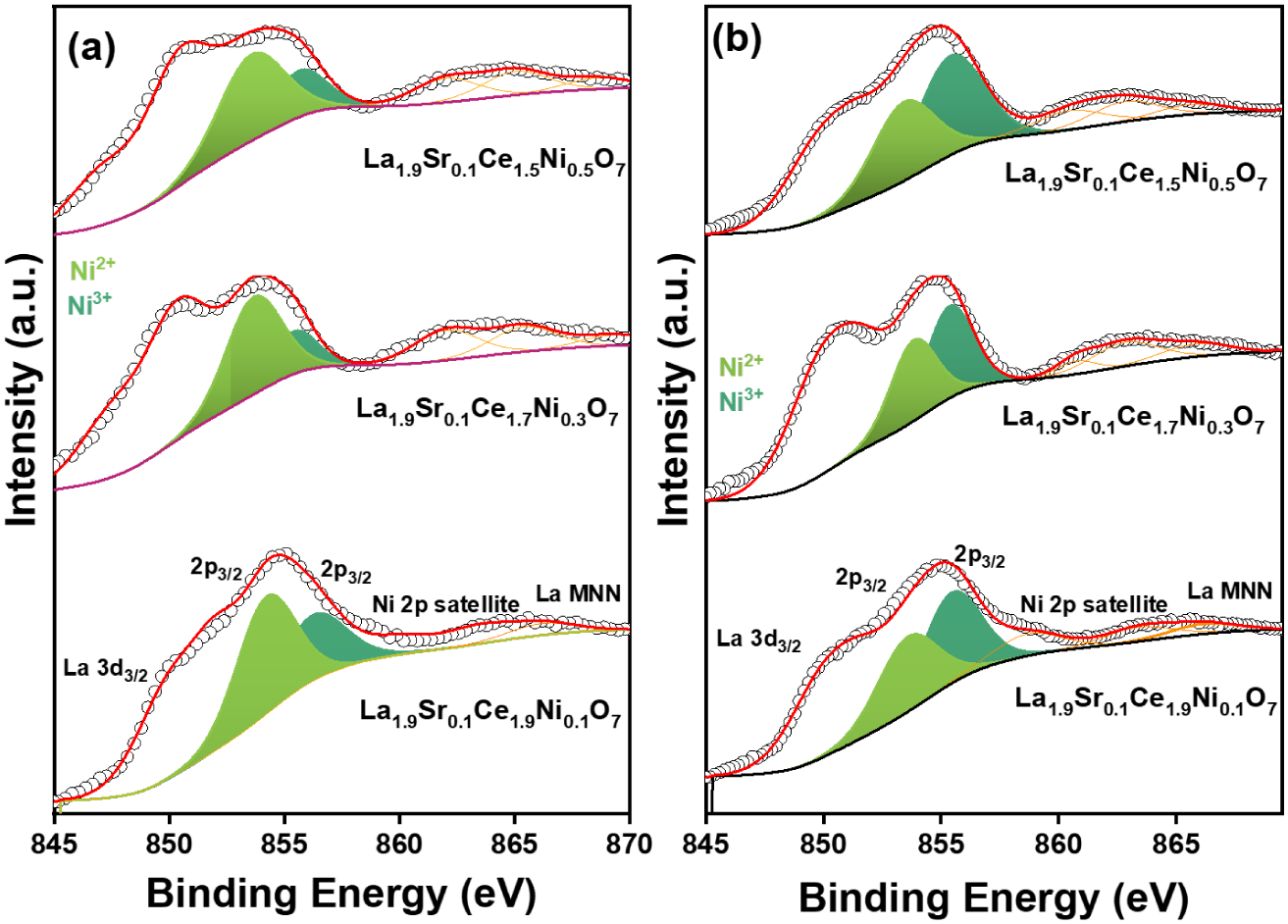
Core-level XPS of Ni 2*p* without La 3*d* in (a) freshly synthesized and (b) time-on-stream exhausted La_1.9_Sr_0.1_Ce_1.9_Ni_0.1_O_7_, La_1.9_Sr_0.1_Ce_1.7_Ni_0.3_O_7_, and La_1.9_Sr_0.1_Ce_1.5_Ni_0.5_O_7_ catalysts.

Figure 7a demonstrates the deconvoluted five peaks with their ten doublets corresponding to Ce^4+^ and Ce^3+^ from the freshly prepared catalysts. Ce^4+^ splits into u–v (882.18–900.18 eV), u″–v″ (888.15–907.59 eV), and u‴–v‴ (896.98–915.67 eV), corresponding to the final states configuration of Ce^4+^ 3*d* ^9^4*f* ^2^O2*p* ^4^, Ce^4+^ 3*d* ^9^4*f* ^1^O2*p* ^5^, and Ce^4+^ 3*d* ^9^4*f* ^0^O2*p* ^6^, respectively. The peaks at binding energies of u^o^–v^o^ (881.6–899.3 eV) and u′–v′ (884.7–903.2 eV) indicate the Ce^3+^ oxidation state arising from electronic states of Ce^3+^ 3*d* ^9^4*f* ^2^O2*p* ^5^ and Ce^3+^ 3*d* ^9^4*f* ^1^O2*p* ^6^, respectively. Upon aliovalent doping of Sr and Ni, the relative surface concentration of Ce^3+^ and Ce^4+^ changed in the as-prepared materials.^53,54^ Apparently, with initial Sr and Ni lattice substitution in La_1.9_Sr_0.1_Ce_1.9_Ni_0.1_O_7_, there was oxidation of Ce^3+^ to Ce^4+^ to compensate for the formal charge (Table S5). Interestingly, there was no gradual oxidation of Ce^3+^ with higher doping of Ni in the freshly prepared La_1.9_Sr_0.1_Ce_1.7_Ni_0.3_O_7_, and La_1.9_Sr_0.1_Ce_1.5_Ni_0.5_O_7_ corroborating the interstitial substitution of the aliovalent ion. The XPS spectra of the 15 h time-on-stream exhausted catalysts in Figure 7b clearly demonstrate the reduction of Ce^4+^ to Ce^3+^ after the DRM reaction. The maximum reduction of Ce^4+^ in La_1.9_Sr_0.1_Ce_1.7_Ni_0.3_O_7_ and La_1.9_Sr_0.1_Ce_1.5_Ni_0.5_O_7_ supports the oxidation of interstitial Ni^2+^ → Ni^3+^ during DRM indicating a superior metal–support interaction.^55^ The H_2_-TPR studies also corroborate the high reducibility of La_1.9_Sr_0.1_Ce_1.7_Ni_0.3_O_7_ and La_1.9_Sr_0.1_Ce_1.5_Ni_0.5_O_7_. Ni^3+^ at the interstitial site may act as an electron acceptor, reducing the tetrahedral symmetry of CH_4_ and thereby facilitating activation of the C–H bond. Earlier experimental studies, along with DFT calculations, have already demonstrated that Ni^δ+^ species promote C–H bond dissociation at lower temperatures compared to metallic Ni.^56^ The facile reduction of Ce^4+^ → Ce^3+^ in the defect fluorite support enabled the formation of Ni^3+^ species by accommodating metal-to-support electron transfer from Ni to Ce.^57–60^ For a better understanding, we further carried out the density of states (DOS) analysis of the energy-minimized pristine and Ni-doped oxides. Figure 7c–f demonstrates a colossal decrease in the gap between conduction band minima constituting Ce projected DOS (PDOS) and valence band maxima constituting Ni PDOS upon increasing Ni concentration in the doped oxides. The charge density distributions of valence band maxima and conduction band minima are mostly localized on the interstitial Ni atoms (Figure S12). The minimization of the band gap might have helped in the efficient electron transfer from Ni^2+^ to Ce^4+^ during the DRM reaction.

**Figure 7:**
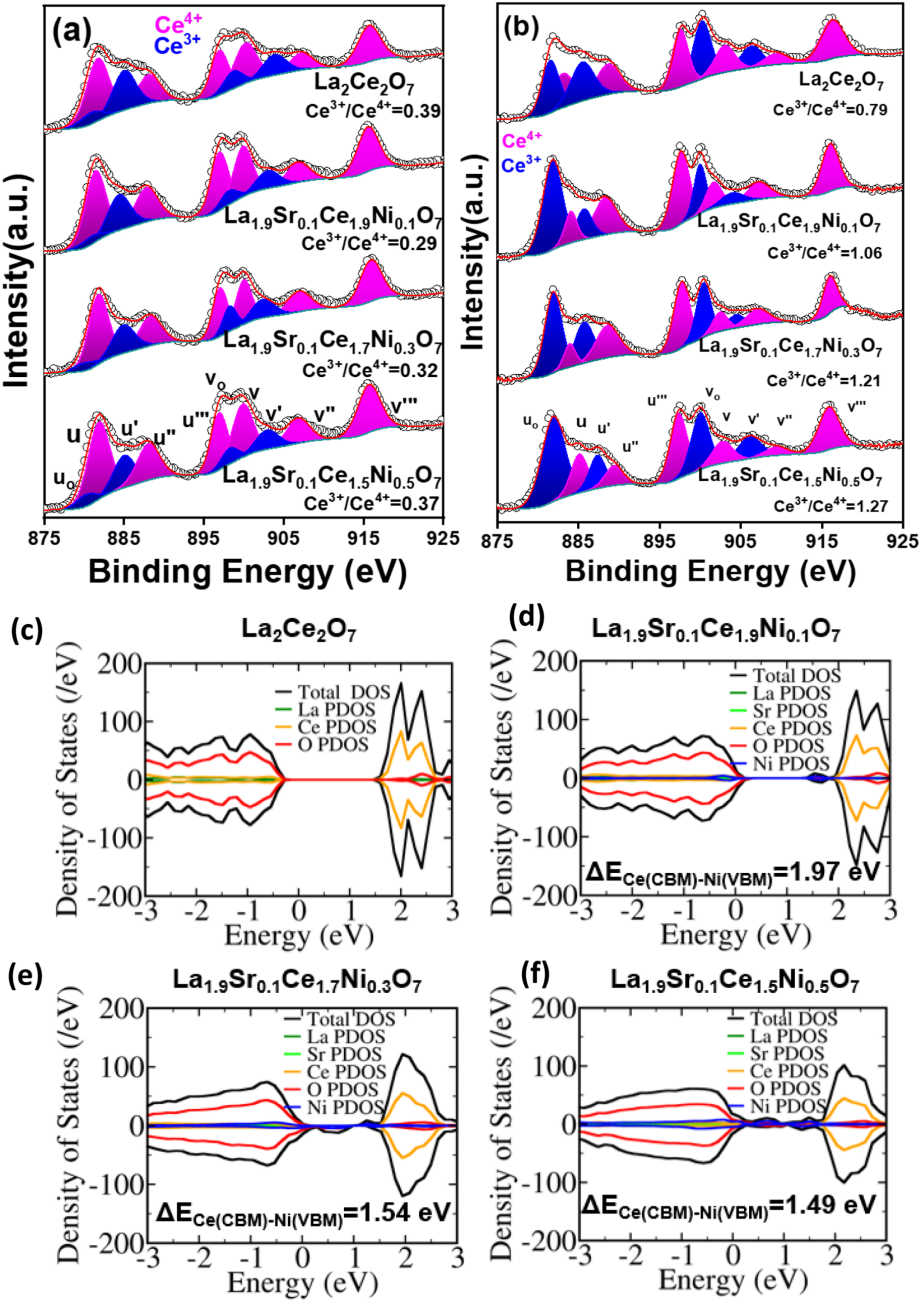
Deconvoluted XPS spectra of Ce 3*d* from (a) freshly prepared and (b) exhausted La_2_Ce_2_O_7_, La_1.9_Sr_0.1_Ce_1.9_Ni_0.1_O_7_, La_1.9_Sr_0.1_Ce_1.7_Ni_0.3_O_7_, and La_1.9_Sr_0.1_Ce_1.5_Ni_0.5_O_7_. (c–f) Projected density of state (PDOS) plots.

Next, we examined the core-level region of O 1*s*. While CH_4_ activation and dehydrogenation occur over Ni^3+^ sites, oxygen vacancies play a crucial role in enhancing the activation and adsorption of CO_2_, thereby facilitating the transformation of the intermediates. Figure S13a presents the deconvoluted XPS spectra of the O 1*s* region for the freshly prepared catalysts. An increase in the percentage of oxygen vacancies in the exhausted catalysts suggests their active involvement in CO_2_ activation through the formation of surface-adsorbed carbonate species during the reaction (Figure S13b). The core-level C 1*s* spectra of the exhausted catalysts also corroborate the formation of carbonate species (Figure S10). The decreased concentration of lattice oxygen (O_L_) in the exhausted catalyst might have been responsible for the low level of coke formation through oxidative C–H activation, as observed experimentally. Therefore, in the following section, we investigate the surface intermediate species formed during DRM in oxygen-vacancy-rich and interstitial Ni^3+^-mediated defect fluorite structures.

The *in situ* FTIR experiments also provided the plausible molecular mechanism of DRM over the doped La_1.9_Sr_0.1_Ce_1.7_Ni_0.3_O_7_ catalyst surface. An equimolar mixture of CO_2_ and CH_4_ was passed over the catalyst, while the temperature was progressively increased from 400 to 650 °C in 50 °C increments, with FTIR spectra recorded at each step. The spectra are presented in Figure 8a and b, with band assignments listed in Table S6. The appearance of C–H bending vibrations from CH_4_ at 1306 cm^–1^, along with characteristic peaks of CH_3_* and CH_x_* in the 1330–1350 cm^–1^ range, suggests CH_4_ dissociation on the catalyst surface.^55^ The presence of oxygenated intermediate species such as CH_*x*_O (1395–1430 cm^–1^), CHO (1748 cm^–1^), CO (1050 cm^–1^), and HCOO^–^ (1550 cm^–1^) indicates that CH_4_ activation follows an oxidative pathway over La_1.9_Sr_0.1_Ce_1.7_Ni_0.3_O_7_.^3,61^ A band at 1659 cm^–1^, characteristic of asymmetric CO_3_^2–^, suggests the formation of carbonate intermediates from CO_2_ in the feed. Additionally, an OH stretching frequency was observed at 3645 cm^–1^. Notably, the intensity of these bands increased with rising temperature. The *in situ* FTIR analysis under RWGS conditions over La_1.9_Sr_0.1_Ce_1.7_Ni_0.3_O indicates the formation of CO and H_2_O with time (Figure S14). This rationalizes the significant occurrence of the RWGS reaction under DRM conditions, explaining the lowered H_2_/CO ratio despite the high conversion of the feed reactants. The oxidative dissociation pathway of CH_4_, illustrated in Figure 8c, describes the conversion of Ni^2+^ → Ni^3+^ and low coke formation during DRM in La_1.9_Sr_0.1_Ce_1.7_Ni_0.3_O_7_.

**Figure 8:**
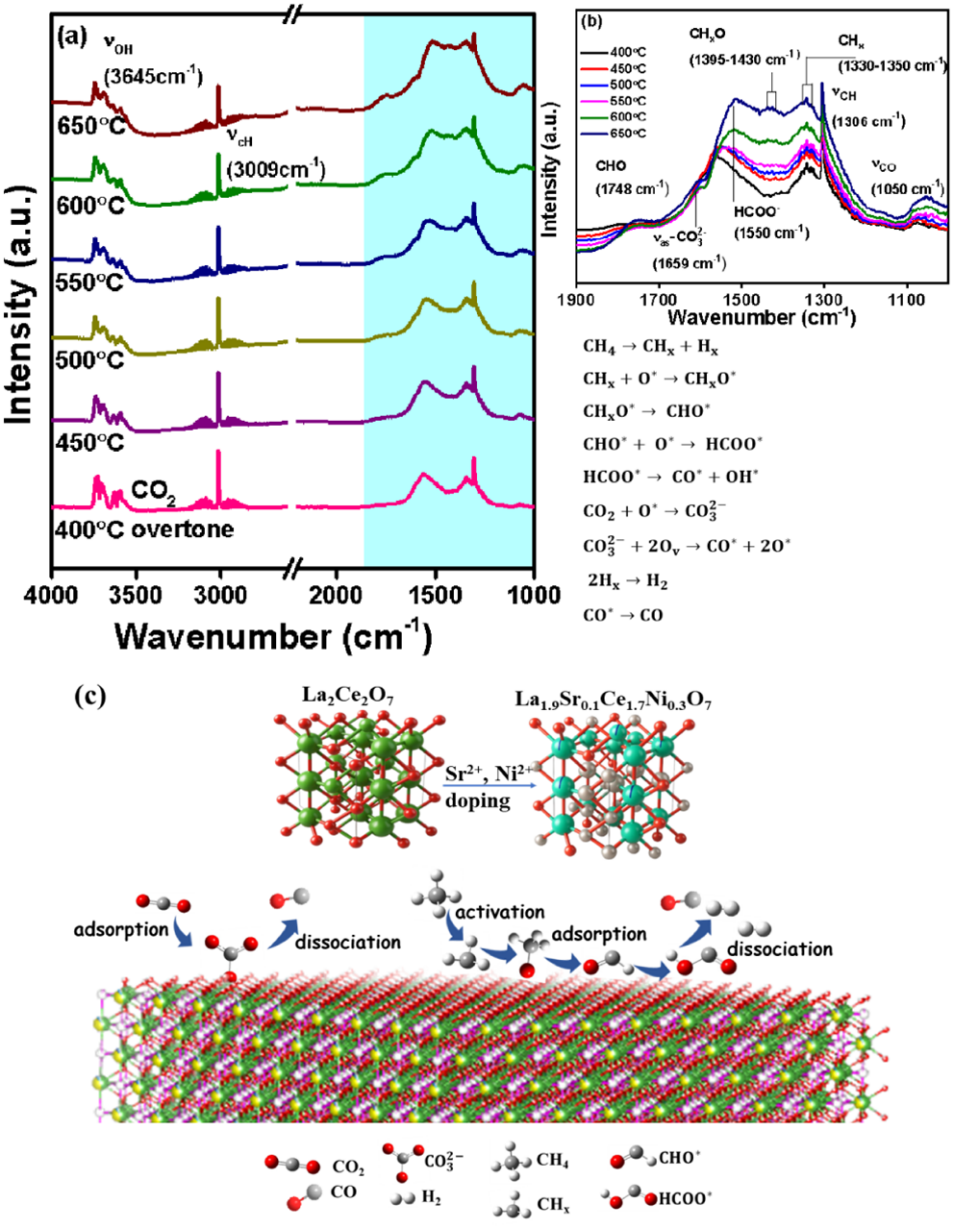
(a) *In situ* FTIR of La_1.9_Sr_0.1_Ce_1.7_Ni_0.3_O_7_ with a feed mixture of CH_4_, CO_2_ and N_2_. (b) An enlarged view and (c) a schematic representation of the DRM mechanism on La_2–*x*_Sr_*x*_Ce_2–*y*_Ni_*y*_O_7_.

## 4 Conclusions

The solution combustion synthesis produced defect fluorites of La_2–*x*_Sr_*x*_Ce_2–*y*_Ni_*y*_O_7−δ_. Detailed structural analysis using powder XRD, Rietveld refinement, EPR, and Raman spectroscopy revealed that while Ni substituted Ce at the Wyckoff 16*c* site in La_1.9_Sr_0.1_Ce_1.9_Ni_0.1_O_7_, it occupied interstitial sites in La_1.9_Sr_0.1_Ce_1.7_Ni_0.3_O_7_ and La_1.9_Sr_0.1_Ce_1.5_Ni_0.5_O_7_. These findings were further corroborated by DFT calculations. A minor presence of LaNiO_3_ was also observed with high Ni content. Electron micrographs indicated that the synthesized materials had an average particle size of approximately 25 nm. Comprehensive catalytic DRM studies demonstrated that the presence of Ni ions in interstitial sites in La_1.9_Sr_0.1_Ce_1.7_Ni_0.3_O_7_ and La_1.9_Sr_0.1_Ce_1.5_Ni_0.5_O_7_, coupled with lattice strain, played a crucial role in enhancing reforming activity by lowering activation energies and increasing the TOF. The presence of the minor LaNiO_3_ phases might have also influenced the catalytic performances. Both La_1.9_Sr_0.1_Ce_1.7_Ni_0.3_O_7_ and La_1.9_Sr_0.1_Ce_1.5_Ni_0.5_O_7_ exhibited stable performance over a 50 h time-on-stream study, achieving over 70% CH_4_ conversion and 83% CO_2_ conversion, with an H_2_/CO ratio exceeding 0.7. TGA analysis indicated minimal coke formation with an average deposition rate of 11 and 12 μg_c_ g_cat_^–1^ h^–1^ for La_1.9_Sr_0.1_Ce_1.7_Ni_0.3_O_7_ and La_1.9_Sr_0.1_Ce_1.5_Ni_0.5_O_7_, respectively. H_2_-TPR studies revealed a significant improvement in catalyst reducibility at lower temperatures with increasing Ni doping, with H_2_ uptake values of 694.7 and 660.4 μmol/g for La_1.9_Sr_0.1_Ce_1.7_Ni_0.3_O_7_ and La_1.9_Sr_0.1_Ce_1.5_Ni_0.5_O_7_, respectively. CO_2_-TPD studies further confirmed an increase in medium and strong basic sites with higher Ni doping. *Ex situ* XPS studies demonstrated that Ce^4+^ was reduced, while Ni^2+^ was oxidized after the time-on-stream DRM reaction. These results suggest that the facile reduction of Ce^4+^ → Ce^3+^ in the defect fluorite support facilitated the oxidation of Ni^2+^ → Ni^3+^ through metal-to-support electron transfer from Ni to Ce. DFT analysis indicated that a reduction in the band gap between Ce and Ni PDOS likely promoted efficient electron transfer from Ni^2+^ to Ce^4+^ during DRM. *In situ* FTIR studies further elucidated the oxidative dissociation of CH_4_ over the defect fluorite catalyst. This comprehensive study offers valuable insights into the role of defect engineering in oxide catalysts, providing a foundation for the development of advanced materials for dry methane reforming.

## Supplementary Material



## Notes

Elemental composition from XRF and ICP-OES; structural properties of the catalysts; powder XRD patterns; refinement parameters; SEM, SAED, HAADF-STEM; STY of H_2_ and CO; variation of fractional conversions; activation energy data; CO-pulse study; coke comparison data; survey photoelectron spectra; charge density distribution; Ni^2+^/Ni^3+^ and Ce^3+^/Ce^4+^ ratios; H_2_-TPR, CO_2_-TPD, XPS, and *in situ* FTIR data (PDF)
