# Supplementary material for: La2–x Sr x Ce2–y Ni y O7 Catalysts with Interstitial Nickel for Enhanced Dry Reforming of Methane
Source: ACS Appl Mater Interfaces. 2025 Oct 30;17(45):61941–54. doi: 10.1021/acsami.5c11404 (PMC12616595; doi:10.1021/acsami.5c11404)
Supplement: Supplementary file 1 [file am5c11404_si_001.pdf]

# Supplementary Information

# $La_{2-x}Sr_xCe_{2-y}Ni_yO_7$ Catalysts with Interstitial Nickel for Enhanced Dry Reforming of Methane

**Aathira Bhaskaran,[a] Naga Pranava Sree Kothoori,[b] Pralok K. Samanta,[a] Stéphane Loridant,[c] Patrick Da Costa,[d] Satyapaul A Singh,[e,f] Sounak Roy*[a,f]**

[a]*Department of Chemistry, Birla Institute of Technology and Science (BITS) Pilani, Hyderabad Campus, Hyderabad-500078, India*
[b]*Department of Chemistry, School of Science, Gandhi Institute of Technology and Management (GITAM), Hyderabad-502329, India*
[c]*Université Claude Bernard Lyon 1, CNRS, IRCELYON, UMR 5256, Villeurbanne, F-69100, France*
[d]*Institut Jean le Rond d'Alembert, Sorbonne Université, CNRS UMR 7190, 2 place de la gare de ceinture, 78210 Saint Cyr l'Ecole, France*
[e]*Department of Chemical Engineering, Birla Institute of Technology and Science (BITS) Pilani, Hyderabad Campus, Hyderabad-500078, India*
[f]*Materials Centre for Sustainable Energy and Environment (McSEE), Birla Institute of Technology and Science (BITS) Pilani, Hyderabad Campus, Hyderabad-500078, India*

*Corresponding Author
Sounak Roy
Email: sounak.roy@hyderabad.bits-pilani.ac.in
ORCID ID: 0000-0003-1070-2068

**Table S1**. Elemental composition from XRF, and ICP-OES

| Compound | Elements | Theoretical wt.% | Observed wt.% (XRF) | Observed wt.% (ICP-OES) |
|---|---|---|---|---|
| $La_2Ce_2O_7$ | La | 49.81 | 49.82 | - |
| | Ce | 50.21 | 50.27 | - |
| $La_{1.9}Sr_{0.1}Ce_{1.9}Ni_{0.1}O_7$ | La | 48.42 | 47.97 | 48.12 |
| | Sr | 1.62 | 1.88 | 1.95 |
| | Ce | 48.91 | 49.05 | 48.73 |
| | Ni | 1.18 | 1.29 | 1.22 |
| $La_{1.9}Sr_{0.1}Ce_{1.7}Ni_{0.3}O_7$ | La | 49.91 | 49.82 | 50.12 |
| | Sr | 1.61 | 1.65 | 1.96 |
| | Ce | 45.16 | 44.91 | 44.92 |
| | Ni | 3.31 | 3.75 | 3.07 |
| $La_{1.9}Sr_{0.1}Ce_{1.5}Ni_{0.5}O_7$ | La | 51.54 | 50.57 | 51.81 |
| | Sr | 1.77 | 2.04 | 1.64 |
| | Ce | 41.02 | 40.57 | 42.49 |
| | Ni | 5.77 | 6.92 | 4.50 |

**Table S2.** Structural properties of the catalysts

| Material | Cell parameter (Å) | Cell volume (Å$^3$) | O Occupancy | $\chi^2$ | Micro strain, $\varepsilon(\times 10^{-3})$ |
|---|---|---|---|---|---|
| $La_2Ce_2O_7$ | 5.58 | 174.28 | 0.86 | 1.15 | 1.81 |
| $La_{1.9}Sr_{0.1}Ce_{1.9}Ni_{0.1}O_7$ | 5.57 | 173.58 | 0.74 | 1.32 | 1.41 |
| $La_{1.9}Sr_{0.1}Ce_{1.7}Ni_{0.3}O_7$ | 5.59 | 175.56 | 0.72 | 1.30 | 2.05 |
| $La_{1.9}Sr_{0.1}Ce_{1.5}Ni_{0.5}O_7$ | 5.61 | 175.72 | 0.67 | 1.58 | 4.13 |

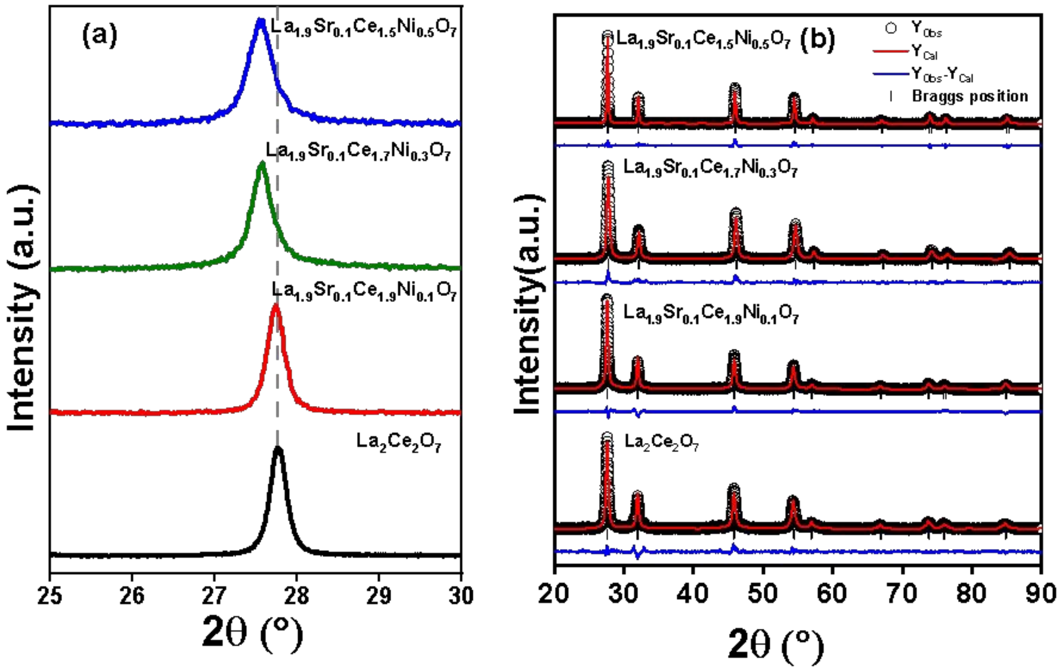


**Figure S1.** (a) shift in the 2θ values (b) Rietveld Refinement for the materials $La_2Ce_2O_7$, $La_{1.9}Sr_{0.1}Ce_{1.9}Ni_{0.1}O_7$, $La_{1.9}Sr_{0.1}Ce_{1.7}Ni_{0.3}O_7$, $La_{1.9}Sr_{0.1}Ce_{1.5}Ni_{0.5}O_7$

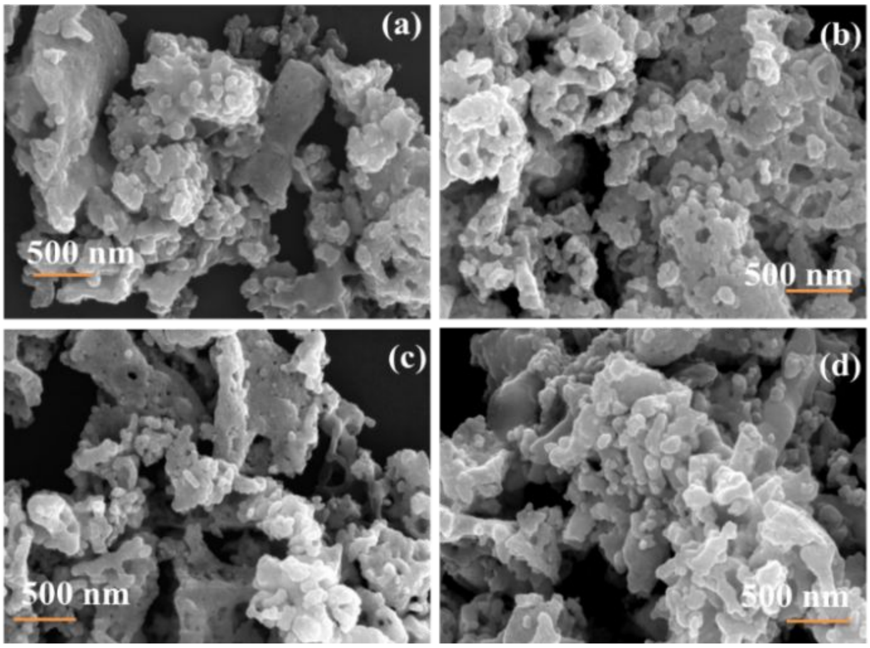


**Figure S2**. SEM images of the fresh catalysts (a) $La_2Ce_2O_7$ (b) $La_{1.9}Sr_{0.1}Ce_{1.9}Ni_{0.1}O_7$ (c) $La_{1.9}Sr_{0.1}Ce_{1.7}Ni_{0.3}O_7$ and (d) $La_{1.9}Sr_{0.1}Ce_{1.5}Ni_{0.5}O_7$

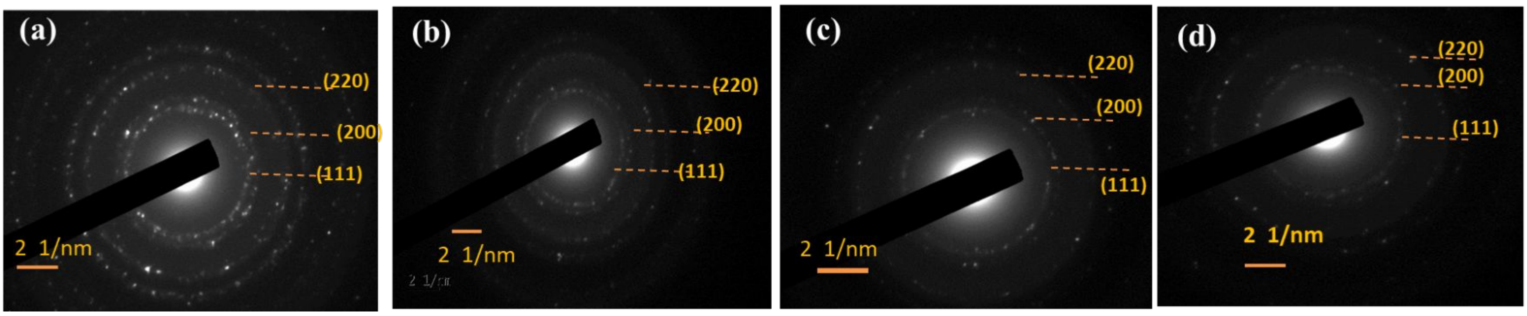


**Figure S3.** SAED pattern of the fresh catalysts (a) $La_2Ce_2O_7$ (b) $La_{1.9}Sr_{0.1}Ce_{1.9}Ni_{0.1}O_7$ (c) $La_{1.9}Sr_{0.1}Ce_{1.7}Ni_{0.3}O_7$ and (d) $La_{1.9}Sr_{0.1}Ce_{1.5}Ni_{0.5}O_7$

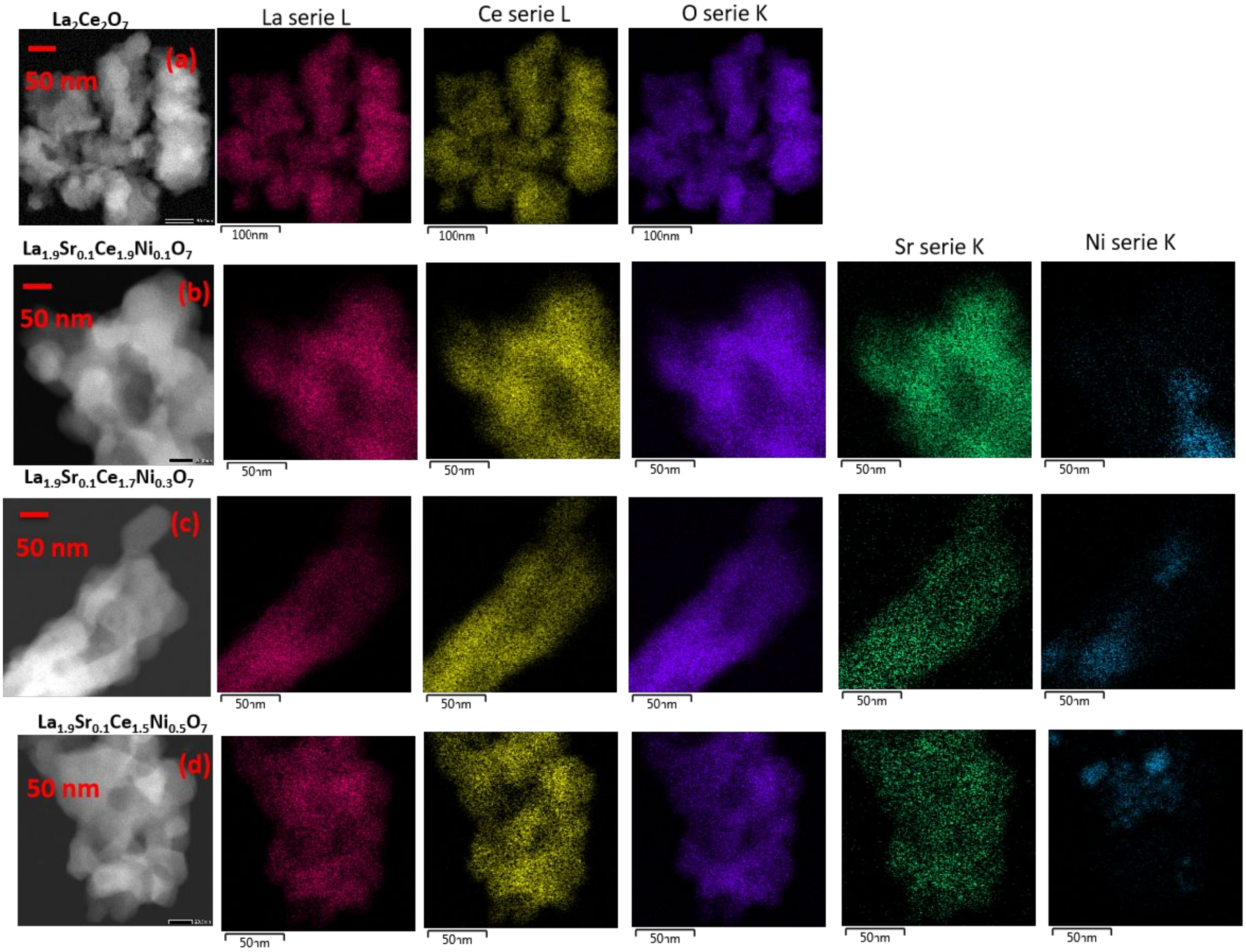


=

**Figure S4**. HAADF-STEM, (elemental mapping) of the fresh catalysts (a) $La_2Ce_2O_7$, (b) $La_{1.9}Sr_{0.1}Ce_{1.9}Ni_{0.1}O_7$, (c) $La_{1.9}Sr_{0.1}Ce_{1.7}Ni_{0.3}O_7$ and (d) $La_{1.9}Sr_{0.1}Ce_{1.5}Ni_{0.5}O_7$

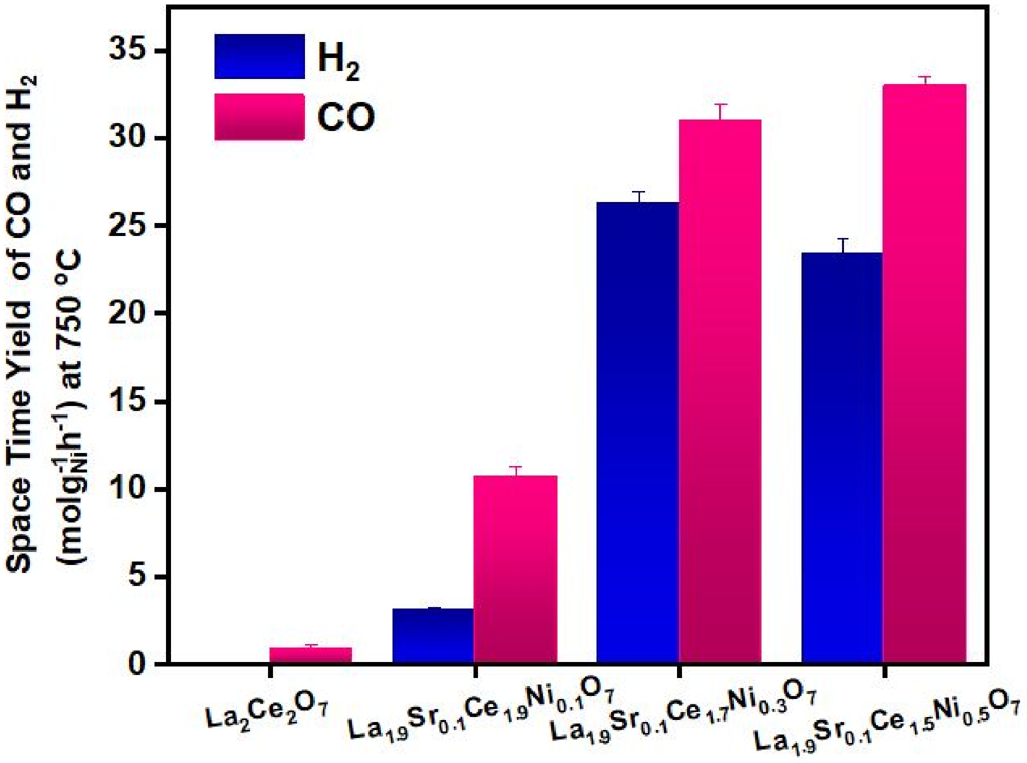


**Figure S5.** STY of $H_2$ and CO normalized with Ni content over the catalysts at 750 $^oC$

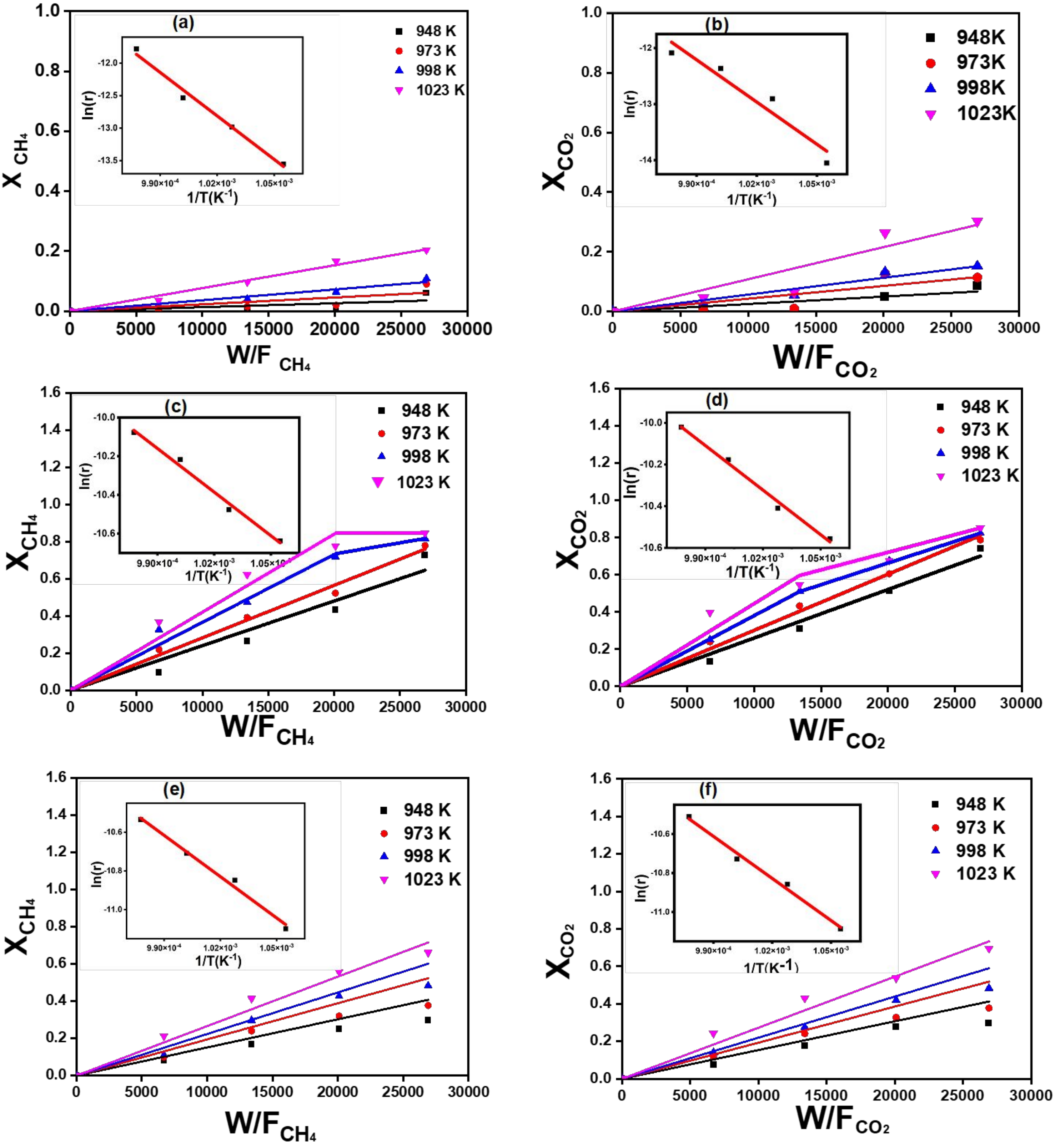


**Figure S6.** Variation of fractional conversions of $CH_4$ and $CO_2$ with W/F (6700, 13400, 20200 and 26910 g μmol$^{-1}$ s) over (a,b) $La_{1.9}Sr_{0.1}Ce_{1.9}Ni_{0.1}O_7$, (c,d) $La_{1.9}Sr_{0.1}Ce_{1.7}Ni_{0.3}O_7$, (e,f) $La_{1.9}Sr_{0.1}Ce_{1.5}Ni_{0.5}O_7$. Arrhenius plots are in the inset as ln(r) vs. 1/T (K$^{-1}$).

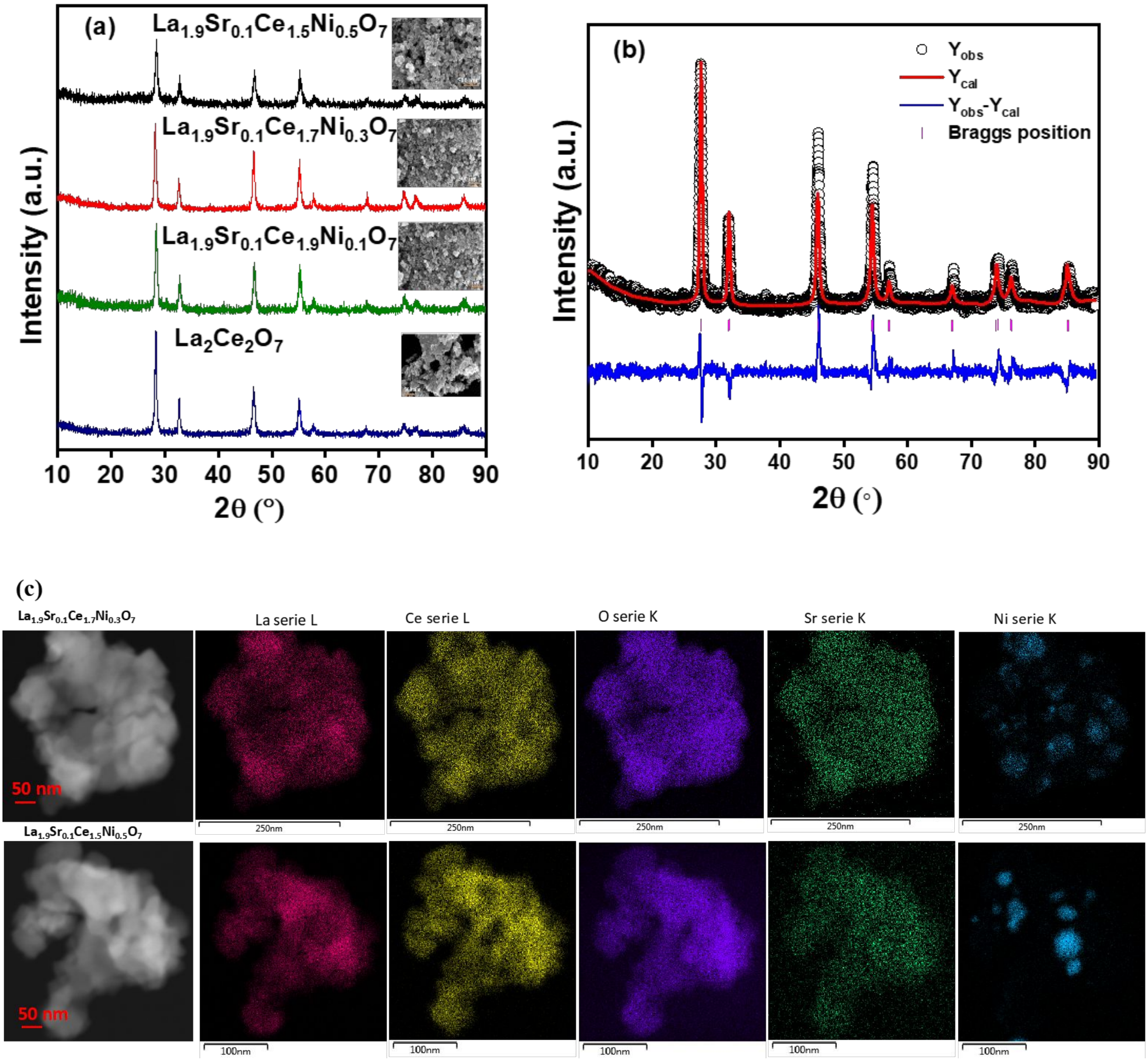


**Figure S7. (a)** Powder XRD and FE-SEM of the 5 hours exhausted $La_2Ce_2O_7$, $La_{1.9}Sr_{0.1}Ce_{1.9}Ni_{0.1}O_7$, $La_{1.9}Sr_{0.1}Ce_{1.7}Ni_{0.3}O_7$ and $La_{1.9}Sr_{0.1}Ce_{1.5}Ni_{0.5}O_7$ (b) Rietveld Refinement of exhausted $La_{1.9}Sr_{0.1}Ce_{1.7}Ni_{0.3}O_7$ after 50 hours and (c) HAADF-STEM, (elemental mapping) of the exhausted catalysts $La_{1.9}Sr_{0.1}Ce_{1.7}Ni_{0.3}O_7$ and $La_{1.9}Sr_{0.1}Ce_{1.5}Ni_{0.5}O_7$.

**Table S3.** Coke comparison study

| **Catalyst** | **Preparation method** | **Condition (temp., time)** | **Conversion ($CH_4$: $CO_2$)** | **Weight loss due to coke formation** | **Ref.** |
|---|---|---|---|---|---|
| $Pt/Mg_{1-x}Ni_xO$ | co-precipitation method | 900 °C<br>10 hr | 82%<br>98% | 6% | 1 |
| Core-shell 12%$Ni@Al_2O_3$ | inverse microemulsion method | 800 °C<br>50 hr | ~90%<br>~95% | 15% | 2 |
| Na, K, and Cs $Ni/ZrO_2$ | incipient wetness impregnation | 650 °C<br>12 hr | 50%<br>60% | 3% | 3 |
| $MgO$-$Al_2O_3$ | sequential incipient wetness impregnation | 800 °C<br>80hr | 90%<br>95% | 11.7% | 4 |
| spherical hollow $Ni/SiO_2$ | modified Stober ¨ method | 750 °C | 70%<br>80 % | 30% | 5 |
| $LaNiO_3/Al_2O_3$ | combustion method | 800 °C<br>24hr | 60%<br>70% | 7.6% | 6 |
| $Ni/Ce_{0.9}Eu_{0.1}O_{1.95}$ | hydrothermal method | 600 °C<br>11.6 hr | 33%<br>21% | 18.7% | 7 |
| $Mg_{0.80}Ni_{0.20}Al_2O_4$ | sol-gel method | 700 °C<br>13.4 hr | 72.6%<br>80.7% | 7.8% | 8 |
| 6% $Ni/Al_2O_3$ nanofiber (F) | Incipient wetness impregnation | 750 ◦C<br>10 h, | 83%<br>81% | 12% | 9 |
| $LaMg_{0.2}Fe_{0.2}Co_{0.2}Ni_{0.2}Cu_{0.2}O_3$ | Sol-gel synthesis | 750 °C,<br>25 hr. | 66%,<br>56% | 0.5 | 10 |
| $La_2NiO_4$ | Solution combustion | 750 °C<br>15 hr | 16%<br>29% | 4.5% | 11 |
| $La_{0.95}Ce_{0.05}NiO_3$ | Amorphous citrate decomposition | 750 °C<br>30 hr | 50.5%<br>60% | NA | 12 |
| $LaNi_{0.8}Cu_{0.2}O_3$ | Amorphous citrate decomposition | 800 °C<br>100 hr | 97%<br>99% | NA | 13 |
| $0.5Rh/ZrO_2$ | wetness impregnation | 700 °C<br>30h | 53<br>70 | Negligeble coke | 14 |
| $Ce_{0.70}La0_{.20}Ni_{0.10}O_{2-\delta}$ | combustion method | 750 °C<br>50 hr. | 71%<br>79% | 0.2% | 15 |
| $La_{1.9}Sr_{0.1}Ce_{1.7}Ni_{0.3}O_7$ | Solution combustion | 700<br>50 h | 69.5%<br>72.4% | 3.1% | This work |
| $La_{1.9}Sr_{0.1}Ce_{1.5}Ni_{0.5}O_7$ | Solution combustion | 700<br>50 h | 83.5%<br>86% | 2.2% | This work |

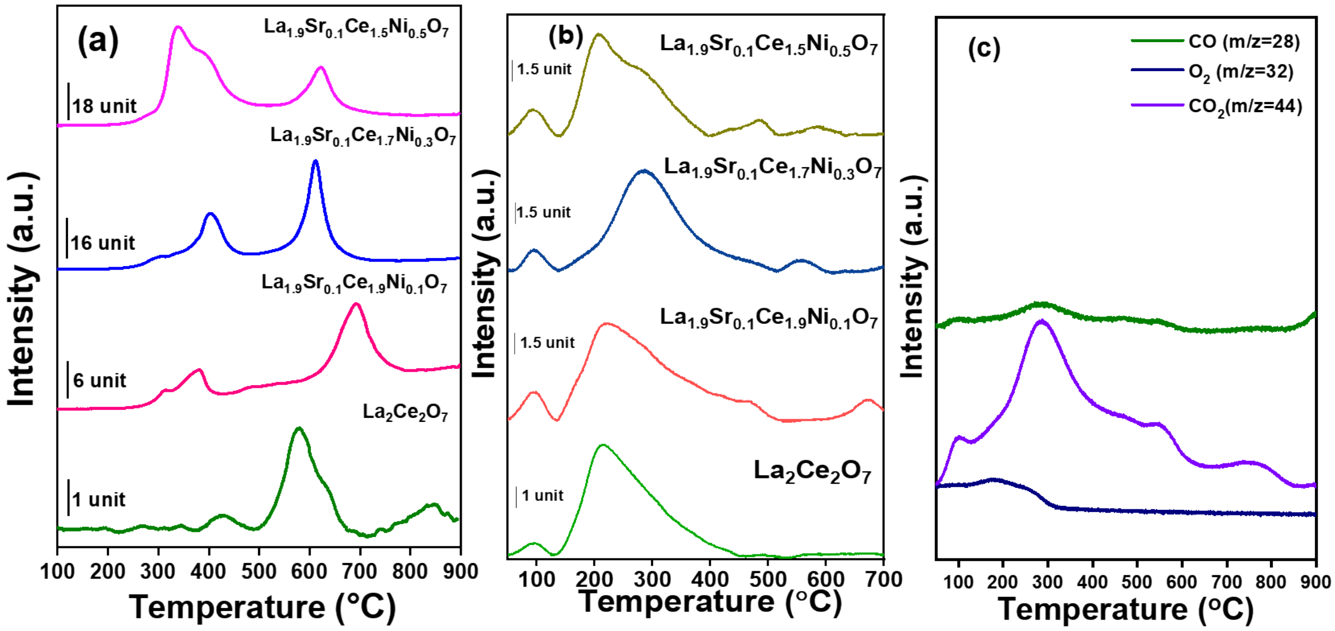


**Figure S8**. (a) $H_2$-TPR, (b) $CO_2$-TPD profiles of $La_2Ce_2O_7$, $La_{1.9}Sr_{0.1}Ce_{1.9}Ni_{0.1}O_7$, $La_{1.9}Sr_{0.1}Ce_{1.7}Ni_{0.3}O_7$, and $La_{1.9}Sr_{0.1}Ce_{1.5}Ni_{0.5}O_7$ and (c) $CO_2$-TPD mass spectra signal of $La_{1.9}Sr_{0.1}Ce_{1.7}Ni_{0.3}O_7$

**Table S4.** $H_2$ consumption and basic sites analysis from $H_2$-TPR and $CO_2$-TPD

| **Catalyst** | **Total $H_2$ consumption (µmol/g)** | **$CO_2$ uptake (µmol/g)** | | | |
|---|---|---|---|---|---|
| | | **$CO_2$ uptake (Temperature in °C)** | **$CO_2$ uptake (Temperature in °C)** | **$CO_2$ uptake (Temperature in °C)** | **Total $CO_2$ uptake** |
| $La_2Ce_2O_7$ | 164.81 | 6.61 (104.4) | 223.73 (222.4 - 381.3) | - | 230.31 |
| $La_{1.9}Sr_{0.1}Ce_{1.9}Ni_{0.1}O_7$ | 305.66 | 14.43 (118.2) | 240.71 (383.1) | 21.31 (511.6) | 276.42 |
| $La_{1.9}Sr_{0.1}Ce_{1.7}Ni_{0.3}O_7$ | 694.81 | 7.35 (113.5) | 334.34 (302.2) | 1.12 (501.1) 17.91 (573.9) | 360.63 |
| $La_{1.9}Sr_{0.1}Ce_{1.5}Ni_{0.5}O_7$ | 660.47 | 12.51 (114.3) | 247.78 (219.9 - 290.3) | 14.14 (501.3) 15.86 (575.3) | 290.15 |

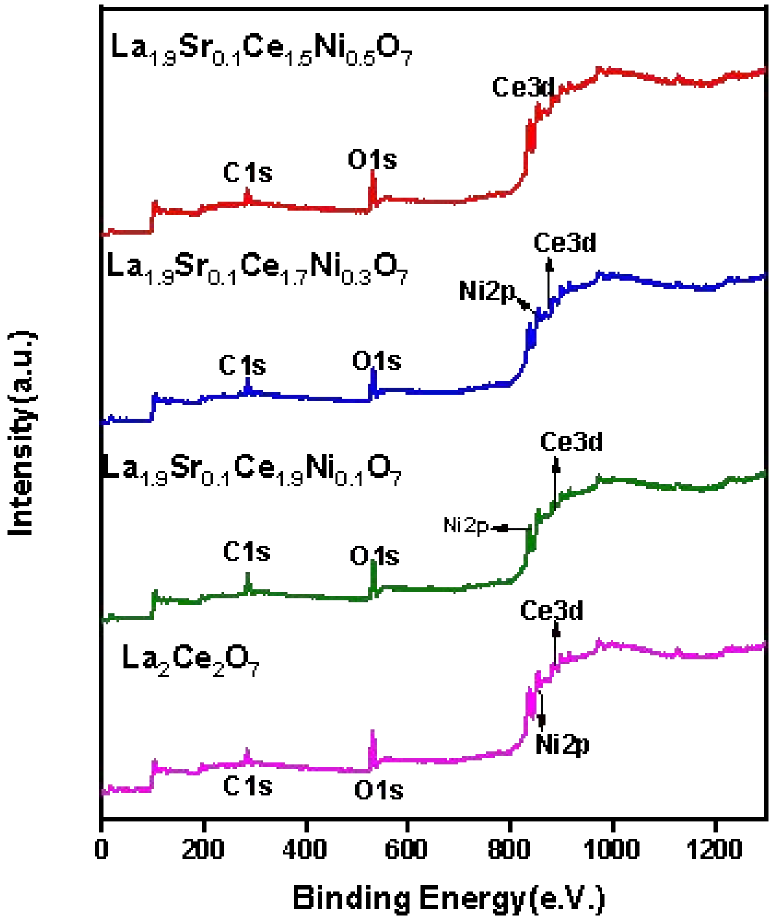


**Figure S9.** Survey photoelectron spectra of $La_2Ce_2O_7$, $La_{1.9}Sr_{0.1}Ce_{1.9}Ni_{0.1}O_7$, $La_{1.9}Sr_{0.1}Ce_{1.7}Ni_{0.3}O_7$, $La_{1.9}Sr_{0.1}Ce_{1.5}Ni_{0.5}O_7$ catalysts

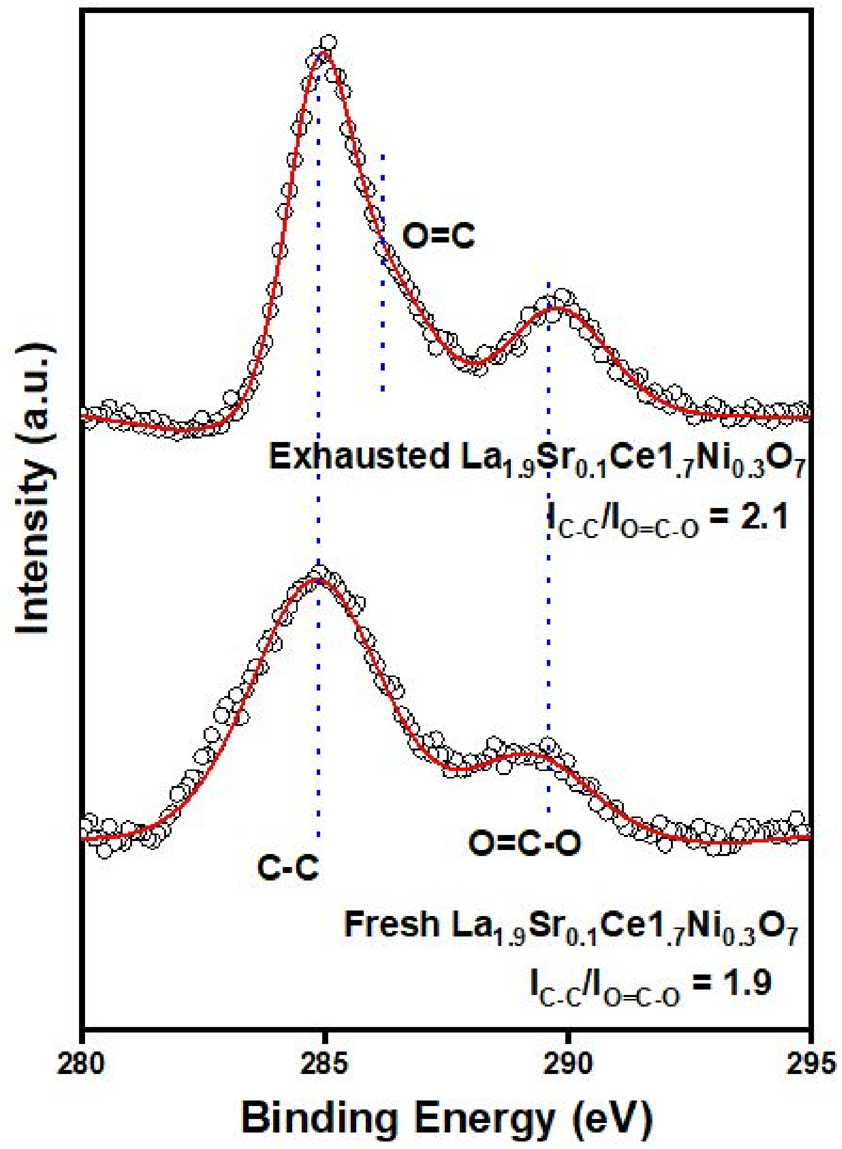


**Figure S10.** XPS core levels of C 1*s* freshly synthesized and time on stream exhausted $La_{1.9}Sr_{0.1}Ce_{1.7}Ni_{0.3}O_7$ catalyst.

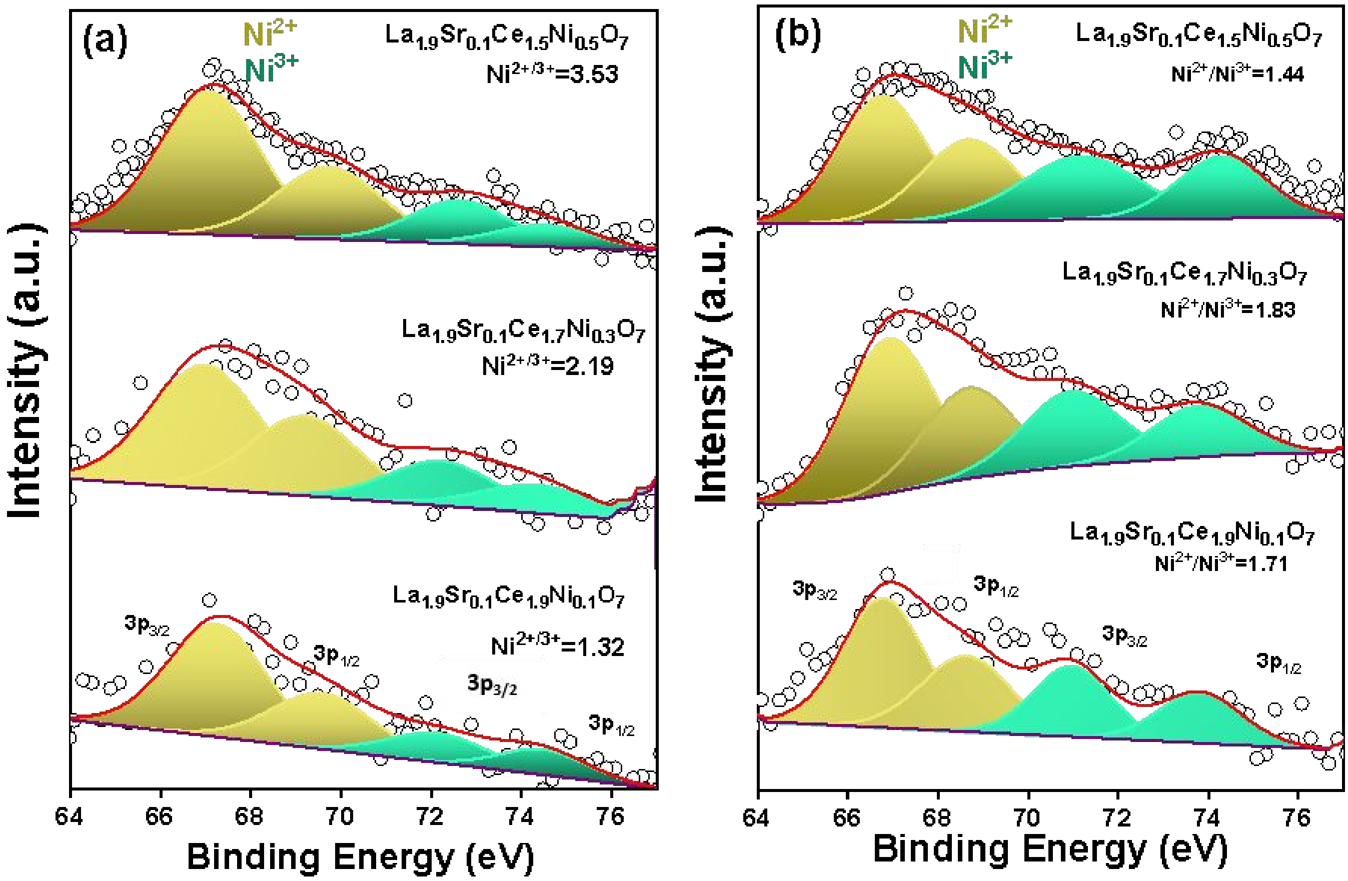


**Figure S11.** Core level XPS of Ni 3*p* in (a) freshly synthesized and (b) time on stream exhausted $La_{1.9}Sr_{0.1}Ce_{1.9}Ni_{0.1}O_7$, $La_{1.9}Sr_{0.1}Ce_{1.7}Ni_{0.3}O_7$, and $La_{1.9}Sr_{0.1}Ce_{1.5}Ni_{0.5}O_7$ catalysts.

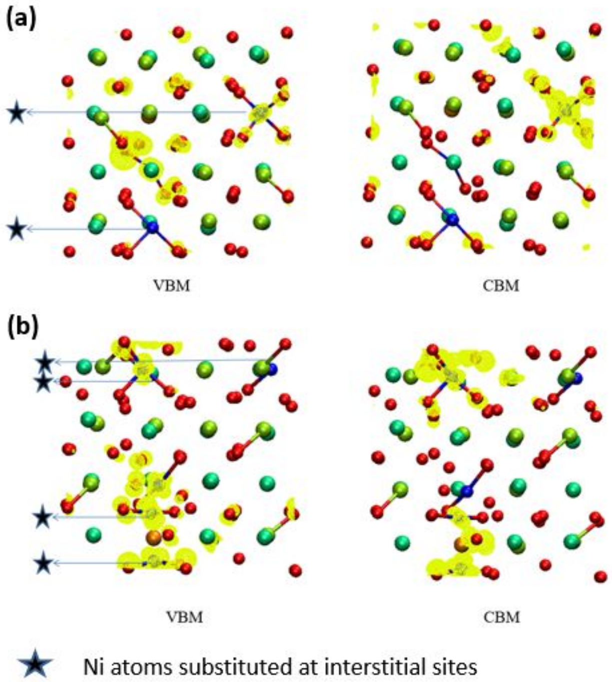


**Figure S12.** Charge density distribution of valence band maxima (VBM) and conduction band minima (CBM) for (a) $La_{1.9}Sr_{0.1}Ce_{1.7}Ni_{0.3}O_7$, and (b) $La_{1.9}Sr_{0.1}Ce_{1.5}Ni_{0.5}O_7$. Color code: Charge density isosurface with isovalue equal to 0.2 (yellow).

**Table S5.** $Ni^{2+}/Ni^{3+}$ and $Ce^{3+}/Ce^{4+}$ ratio before and after the reaction.

| **Catalysts** | **As-prepared $Ce^{3+}/Ce^{4+}$** | **Exhausted $Ce^{3+}/Ce^{4+}$** | **As-prepared $Ni^{2+}/Ni^{3+}$** | **Exhausted $Ni^{2+}/Ni^{3+}$** |
|---|---|---|---|---|
| **$La_2Ce_2O_7$** | 0.39 | 0.79 | | |
| **$La_{1.9}Sr_{0.1}Ce_{1.9}Ni_{0.1}O_7$** | 0.29 | 1.06 | 3.53 | 1.44 |
| **$La_{1.9}Sr_{0.1}Ce_{1.7}Ni_{0.3}O_7$** | 0.32 | 1.21 | 2.19 | 1.83 |
| **$La_{1.9}Sr_{0.1}Ce_{1.5}Ni_{0.5}O_7$** | 0.37 | 1.27 | 1.32 | 1.71 |

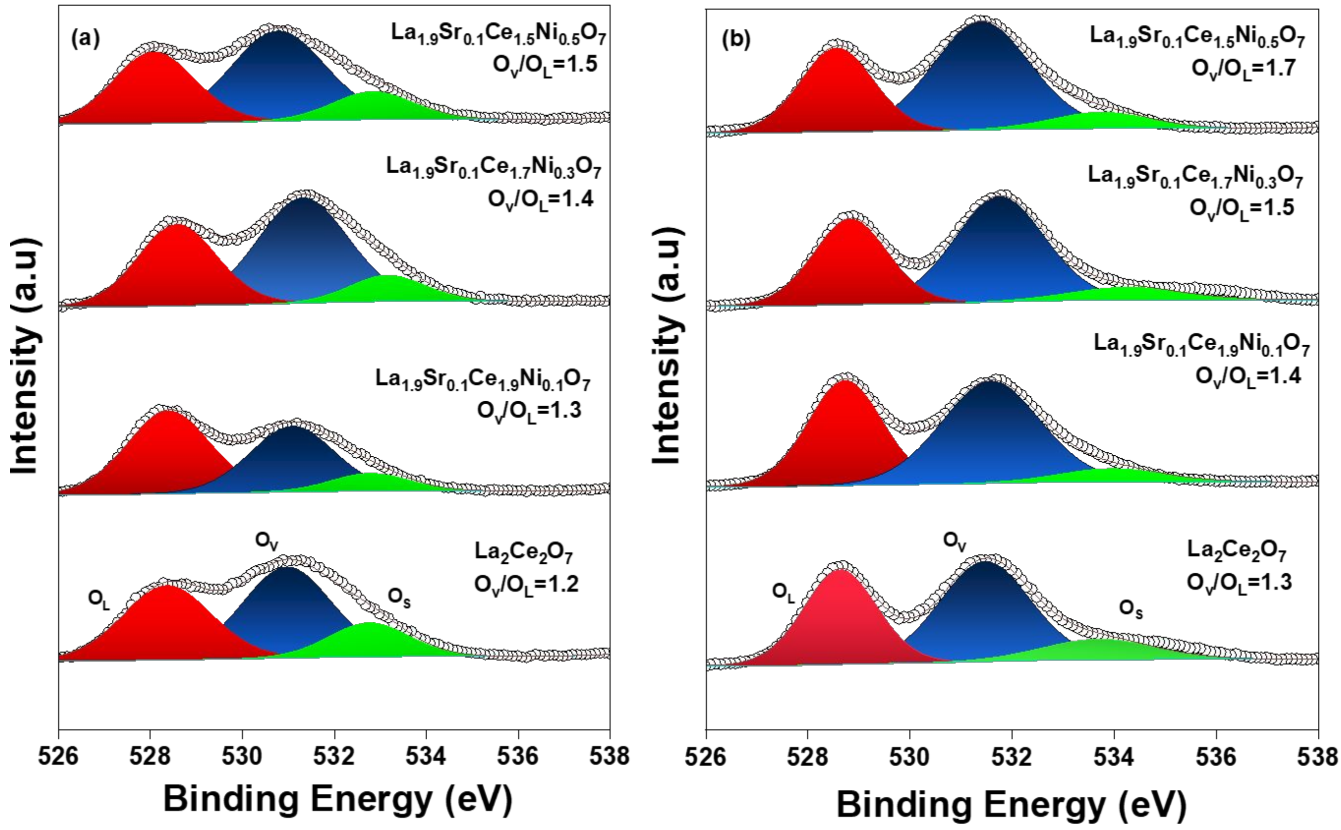


**Figure S13.** Deconvoluted XPS spectra of O 1*s* from (a) freshly prepared and (b) exhausted $La_2Ce_2O_7$, $La_{1.9}Sr_{0.1}Ce_{1.9}Ni_{0.1}O_7$, $La_{1.9}Sr_{0.1}Ce_{1.7}Ni_{0.3}O_7$ and $La_{1.9}Sr_{0.1}Ce_{1.5}Ni_{0.5}O_7$.

**Table S6.** *In situ* FTIR bands of different Intermediate species formed over $La_{1.9}Sr_{0.1}Ce_{1.7}Ni_{0.3}O_7$ with DRM reaction.

| **Reaction condition of DRM** | **Observed Intermediate Species** | **Corresponding wave number ($cm^{-1}$)** | **Ref** |
|---|---|---|---|
| $CO_2$:$CH_4$:$N_2$ 100 ml/min (10:10:80) 400-650 $^oC$ | CO | 1050 | 11 |
| | CH bending for $CH_4$ ($v_4$-CH) | 1306 | 11 |
| | Overtones $CO_2$ | 3580-3732 | 11 |
| | $CH_x^*$ | 1330-1350 | 16 |
| | $CH_xO$ | 1395-1430 | 16 |
| | $HCOO^*$ | 1550 | 16 |
| | CO stretching CHO | 1748 | 17 |
| | $CH_4$ peak ($v_1$-CH) | 3020 | 16 |
| | OH stretching($v_s$-OH) | 3655 | 11 |
| | Asymetric carbonate ($v_{as}$- $CO_3^{2-}$) | 1659 | 18 |

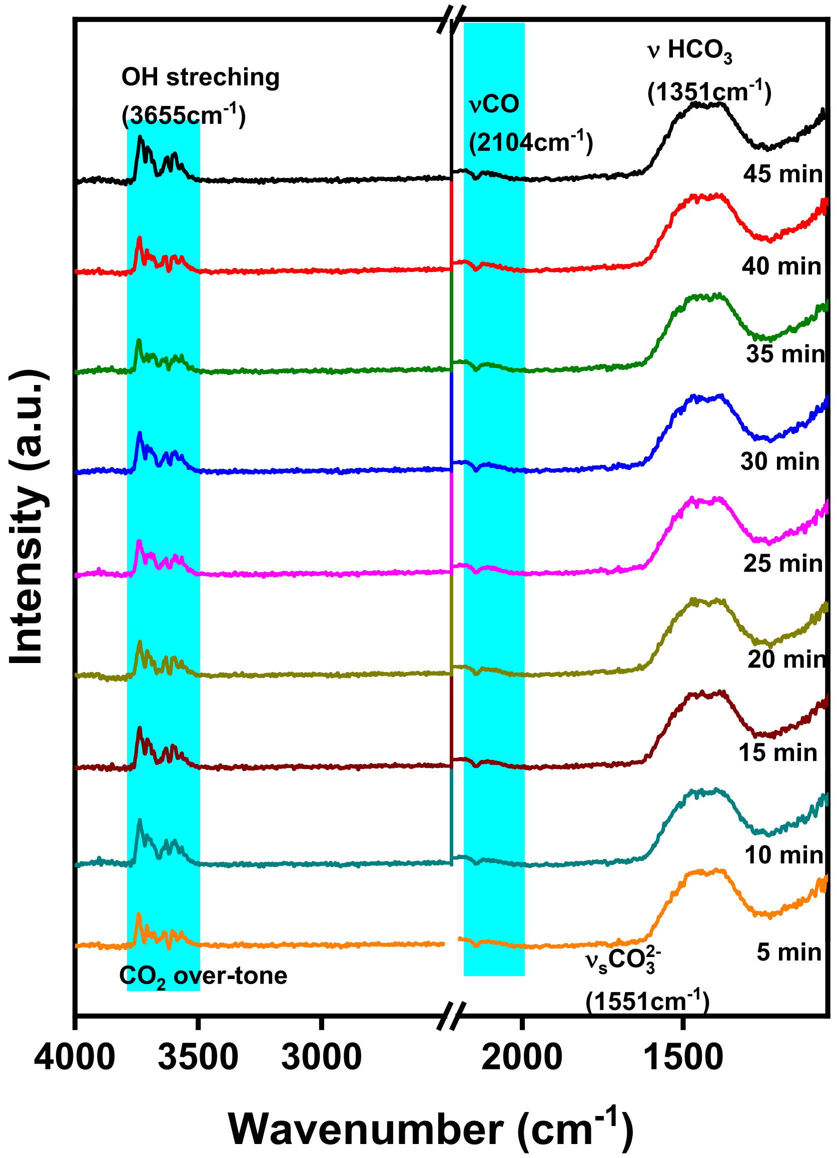


**Figure S14.** *In situ* FTIR of $La_{1.9}Sr_{0.1}Ce_{1.7}Ni_{0.3}O_7$ with a feed mixture of $H_2$, $CO_2$ and $N_2$ at 10:10:80 vol%. under RWGS condition at 700 °C with variation of time.

## References


(1) Al-doghachi, F. A. J.; Islam, A.; Zainal, Z.; Saiman, M. I. High Coke-Resistance Pt / $Mg_{1-x}Ni_xO$ Catalyst for Dry Reforming of Methane. **2016**, 1–22. https://doi.org/10.1371/journal.pone.0145862.

(2) Huang, Q.; Fang, X.; Cheng, Q.; Li, Q.; Xu, X.; Xu, L.; Liu, W.; Gao, Z.; Zhou, W.; Wang, X. Synthesis of a Highly Active and Stable Nickel-Embedded Alumina Catalyst for Methane Dry Reforming: On the Confinement Effects of Alumina Shells for Nickel Nanoparticles. *ChemCatChem* **2017**, *9* (18), 3563–3571. https://doi.org/10.1002/cctc.201700490.

(3) Franz, R.; Franz, R.; Kühlewind, T.; Shterk, G.; Abou-Hamad, E.; Parastaev, A.; Uslamin, E.; Hensen, E. J. M.; Kapteijn, F.; Gascon, J.; Pidko, E. A. Impact of Small Promoter Amounts on Coke Structure in Dry Reforming of Methane over Ni/ZrO2. *Catal. Sci. Technol.* **2020**, *10* (12), 3965–3974. https://doi.org/10.1039/d0cy00817f.

(4) Naidu, B. N.; Kumar, K. D. P. L.; Saini, H.; Kumar, M.; Kumar, T. N.; Prasad, V. V. D. N. Coke Deposition over Ni-Based Catalysts for Dry Reforming of Methane: Effects of $MgO-Al_2O_3$ Support and Ceria, Lanthana Promoters. *J. Environ. Chem. Eng.* **2022**, *10* (1), 106980. https://doi.org/10.1016/j.jece.2021.106980.

(5) Kosari, M.; Askari, S.; Seayad, A. M.; Xi, S.; Kawi, S.; Borgna, A.; Zeng, H. C. Strong Coke-Resistivity of Spherical Hollow $Ni/SiO_2$ Catalysts with Shell-Confined High-Content Ni Nanoparticles for Methane Dry Reforming with $CO_2$. *Appl. Catal. B Environ.* **2022**, *310* (January), 121360. https://doi.org/10.1016/j.apcatb.2022.121360.

(6) Rabelo-Neto, R. C.; Sales, H. B. E.; Inocêncio, C. V. M.; Varga, E.; Oszko, A.; Erdohelyi, A.; Noronha, F. B.; Mattos, L. V. $CO_2$ Reforming of Methane over Supported $LaNiO_3$ Perovskite-Type Oxides. *Appl. Catal. B Environ.* **2018**, *221* (June 2017), 349–361. https://doi.org/10.1016/j.apcatb.2017.09.022.

(7) Wang, Y.; Zhang, R.; Yan, B. $Ni/Ce_{0.9}Eu_{0.1}O_{1.95}$ with Enhanced Coke Resistance for Dry Reforming of Methane. *J. Catal.* **2022**, *407*, 77–89. https://doi.org/10.1016/j.jcat.2022.01.020.

(8) Habibi, N.; Wang, Y.; Arandiyan, H.; Rezaei, M. Effect of Substitution by Ni in $MgAl_2O_4$ Spinel for Biogas Dry Reforming. *Int. J. Hydrogen Energy* **2017**, *42* (38), 24159–24168. https://doi.org/10.1016/j.ijhydene.2017.07.222.

(9) Shen, D.; Huo, M.; Li, L.; Lyu, S.; Wang, J.; Wang, X.; Zhang, Y.; Li, J. Effects of Alumina Morphology on Dry Reforming of Methane over $Ni/Al_2O_3$ Catalysts. *Catal. Sci. Technol.* **2020**, *10* (2), 510–516. https://doi.org/10.1039/c9cy02093d.

(10) Bhaskaran, A.; Singh, S. A.; Roy, S. Exploring La-Based High Entropy Perovskites for Syngas Production via Methane Reforming. *Catal. Lett.* **2025**, *155* (2), 344. https://doi.org/10.1007/s10562-025-05189-0.

(11) Bhaskaran, A.; Singh, S. A.; Da Costa, P.; Roy, S. Unveiling the Catalytic Behaviour of $LaNiO_3$ and $La_2NiO_4$ for Dry Reforming of Methane. *Int. J. Hydrogen Energy* **2024**, *68* (January), 623–634. https://doi.org/10.1016/j.ijhydene.2024.04.295.

(12) Lima, S. M.; Assaf, J. M.; Peña, M. A.; Fierro, J. L. G. Structural Features of $La_{1-x}Ce_xNiO_3$ Mixed Oxides and Performance for the Dry Reforming of Methane. *Appl. Catal. A Gen.* **2006**, *311* (1–2), 94–104. https://doi.org/10.1016/j.apcata.2006.06.010.

(13) Hossain, A.; Ghorai, K.; Bhunia, T.; Llorca, J.; Vasundhara, M.; Bera, P.; Bhaskaran, A.; Roy, S.; Seikh, M.; Gayen, A. Cu-Doped $LaNiO_3$ Perovskite Catalyst for DRM: Revisiting It as a Molecular-Level Nanocomposite. **2024**, 26603–26621. https://doi.org/10.1039/d4cp02252a.

(14) A. Androulakis, I.V. Yentekakis, P. P. Dry Reforming of Methane over Supported Rh and Ru Catalysts: Effect of the Support ($Al_2O_3$, $TiO_2$, $ZrO_2$, YSZ) on the Activity and Reaction Pathway. *Int. J. Hydrogen Energy* **2023**, *48* (87), 33886–33902. https://doi.org/10.1016/j.ijhydene.2023.03.114.

(15) Pino, L.; Italiano, C.; Vita, A.; Laganà, M.; Recupero, V. $Ce_{0.70}La_{0.20}Ni_{0.10}O_{2-\Delta}$ Catalyst for Methane Dry Reforming: Influence of Reduction Temperature on the Catalytic Activity and Stability. *Appl. Catal. B Environ.* **2017**, *218*, 779–792. https://doi.org/10.1016/j.apcatb.2017.06.080.

(16) Wang, H.; Cui, G. Q.; Lu, H.; Li, Z. Y.; Wang, L.; Meng, H.; Li, J.; Yan, H.; Yang, Y. S.; Wei, M. Facilitating the dry reforming of methane with interfacial synergistic catalysis in an Ir@$CeO_{2-x}$ catalyst. Nat. Commun. **2024**, 15, 3765. https://doi.org/10.1038/s41467-024-48122-6.

(17) Chen, C.-H. ; Chen ,H.-K; Huang , W.-H; Chen ,C.-L; Choojun ,K; Sooknoi, T; Tian, H-K; Lin ,Y.-C;Reversal of methanation-oriented to RWGS-oriented Ni/$SiO_2$ catalysts by the exsolution of $Ni^{2+}$ confined in silicalite-1.*Green Chem.*, **2023**, 25, 7582-7597. https://doi.org/10.1039/D3GC02399K.

(18) Köck, E.-M.; Kogler, M.; Bielz, T.; Klötzer, B.; Penner, S. In Situ FT-IR Spectroscopic Study of $CO_2$ and CO Adsorption on $Y_2O_3$, $ZrO_2$, and Yttria-Stabilized $ZrO_2$ J. Phys. Chem. C. **2013**, 117 (34) 17666– 17673 DOI: 10.1021/jp405625x .
